# Supplementary material for: Alternative splicing is coupled to gene expression in a subset of variably expressed genes
Source: bioRxiv. 2023 Oct 11:2023.06.13.544742. Originally published 2023 Jun 14. Preprint. [Version 3] doi: 10.1101/2023.06.13.544742 (PMC10312658; doi:10.1101/2023.06.13.544742)
Supplement: Supplement 1 [file media-1.pdf]

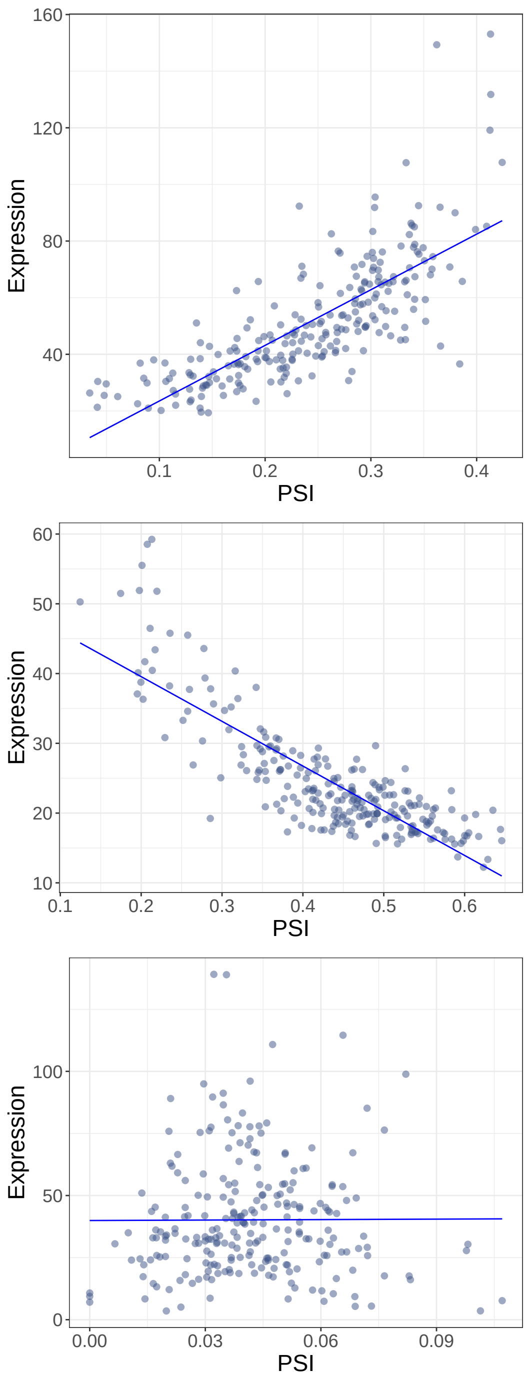

Figure S1: The expression of genes containing UHP (upregulated-high ψ), DHP (downregulated-high ψ), and type 0 exons as a function of the proportion of transcripts including these exons. The expression value and proportions were computed in Spleen. The x-axis shows the proportion of transcript counts for transcripts that include the exon (percent-spliced in, ψ), and the y-axis value is the gene expression or total number of transcript counts for the gene. The genes for the UHP, DHP and type 0 exons displayed in this figure, *CASP8*, *CSTF3* and *ANPEP*, are shown from top to bottom.

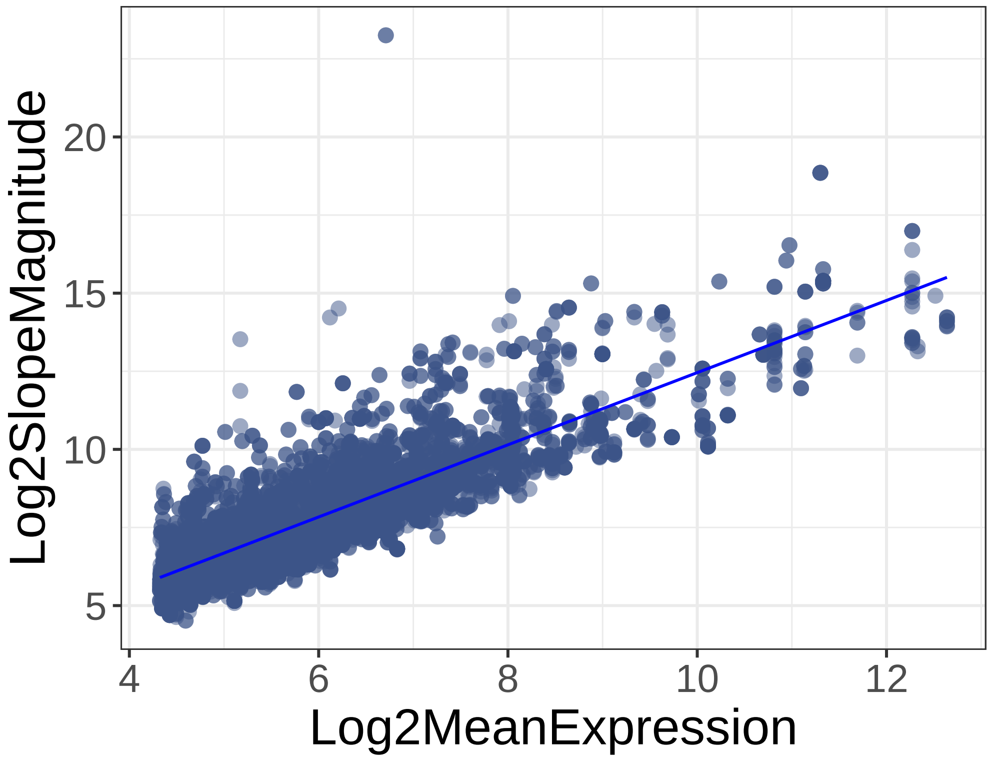

Figure S2: Log_2 **Expression-**ψ **regression slopes vs** log_2 **mean expression.** This Figure the mean expression of genes with at least one UHP or DHP exon (X-axis) with the absolute value of the slope of the the corresponding expression-percent-spliced-in (ψ) regression curve (Y-axis).

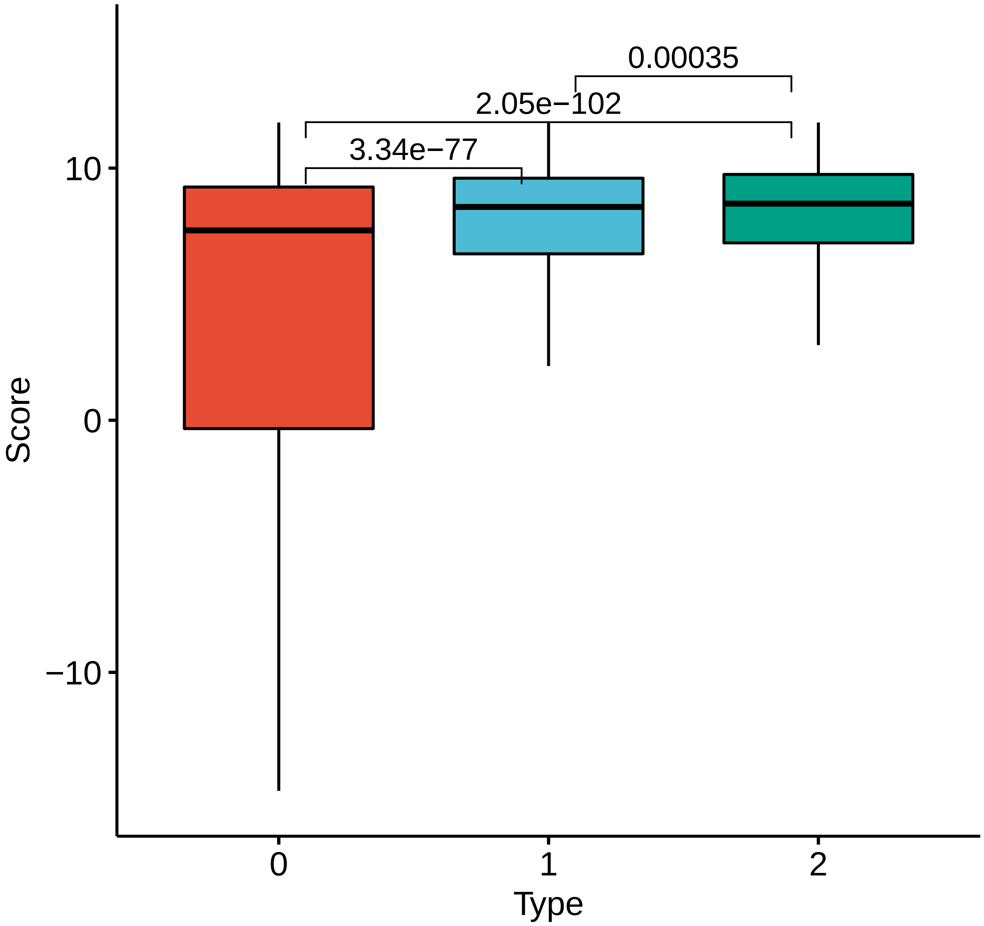

Figure S3: 5’ (donor) splice score distributions. Boxplots illustrate the distribution of the 5’ donor splice score calculated using MaxEntScan (39). 0: type 0 exon; 1: UHP exon; 2: DHP exon.

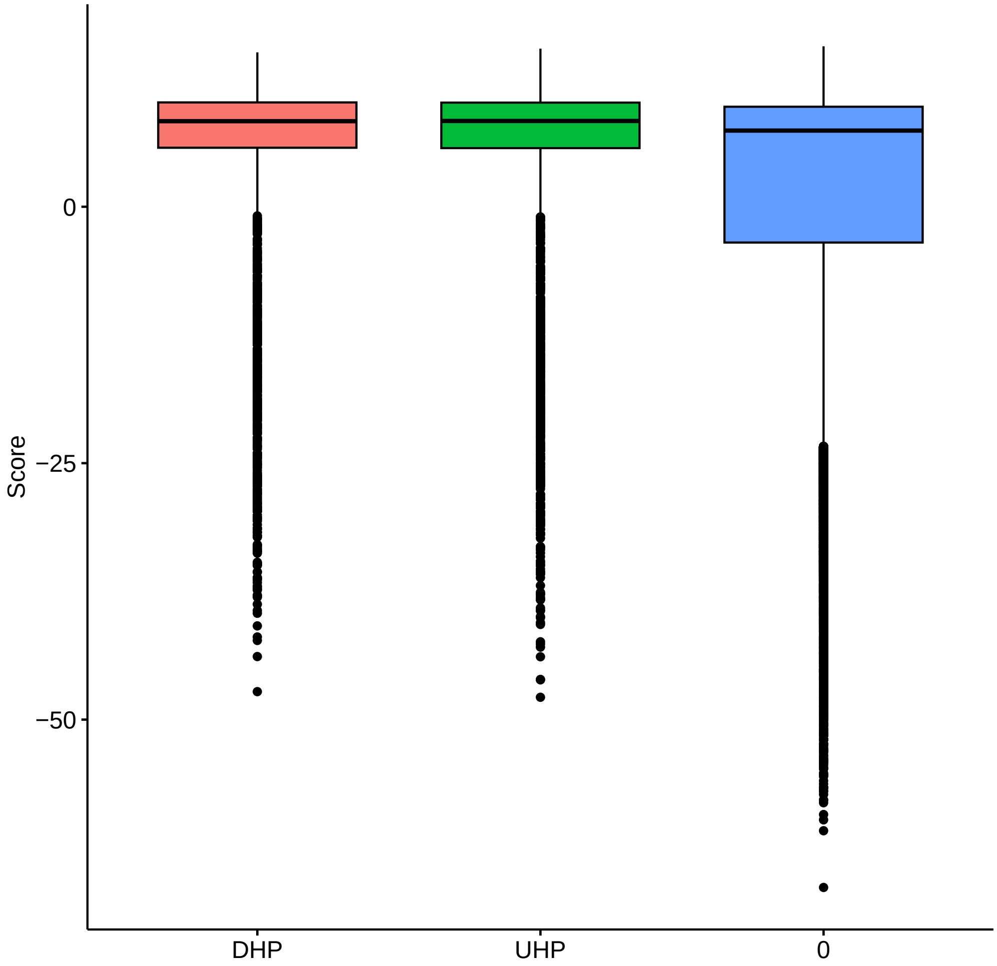

Figure S4: 3' (acceptor) splice score distributions. Boxplots illustrate the distribution of the 3' donor splice score calculated using MaxEntScan (39). The Mann-Whitney p-value of DHP vs type 0 was $p = 2.09^{-43}$, of UHP vs type 0 is $p = 1.19^{-48}$, and UHP vs DHP was $p = 0.95$. 0: type 0 exon; 1: UHP exon; 2: DHP exon.

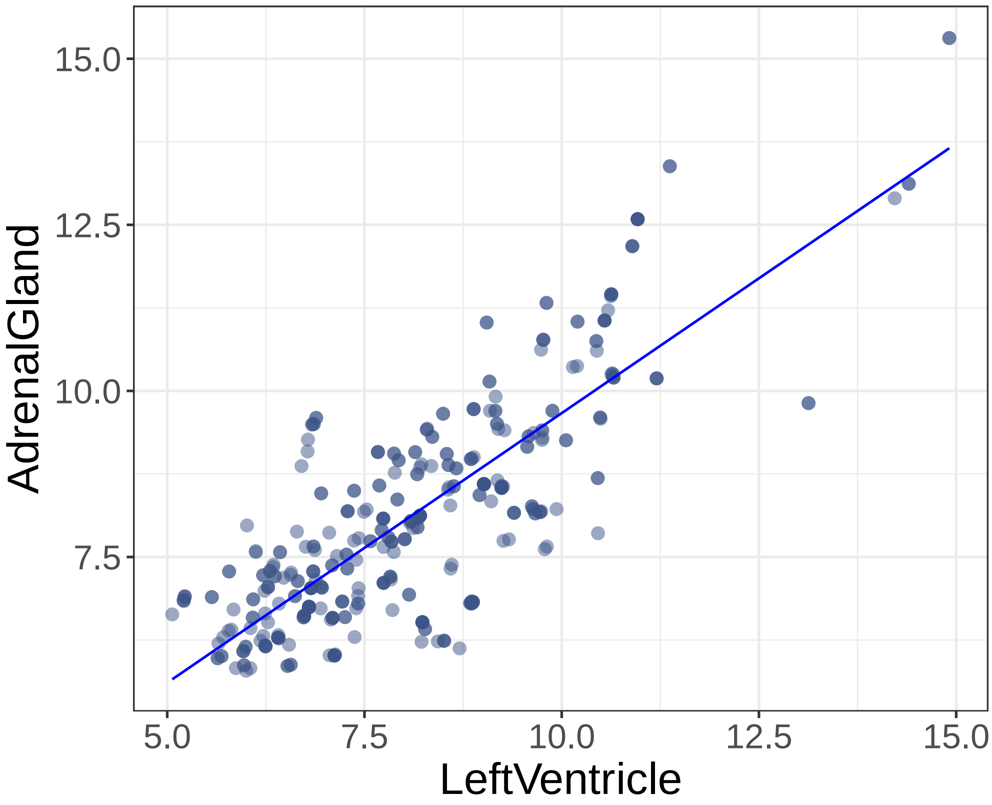

Figure S5: Expression-ψ log2-regression slopes in Heart left ventricle vs adrenal gland. Each point represents the absolute value of the slope of the expression-percent-spliced-in (ψ) regression curves for one UHP or DHP exon.

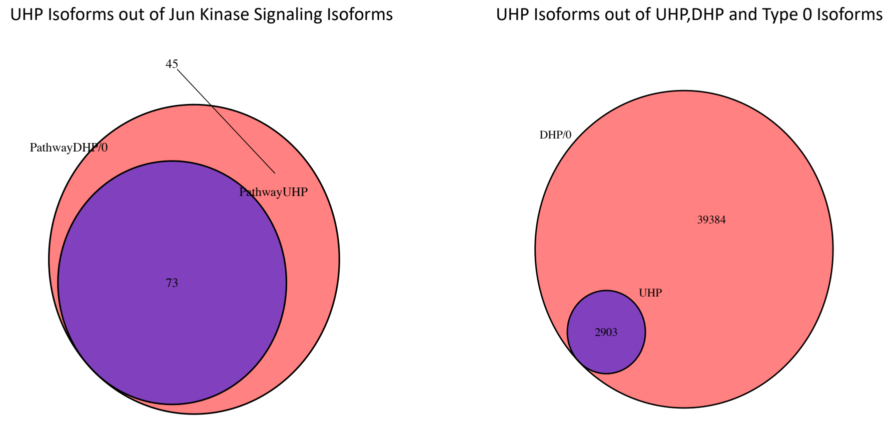

Figure S6: Over-representation UHP-containing isoforms. Proportion of UHP-containing isoforms out of all isoforms belonging to the Jun kinase signaling GO term (left) and the same proportion out of all isoforms containing UHP,DHP or a type 0 exon. The isoforoms are over-represented in the Jun kinase signaling GO term.(Benjamini-Hochberg corrected hyper geometric $p = 1.57^{-51}$)

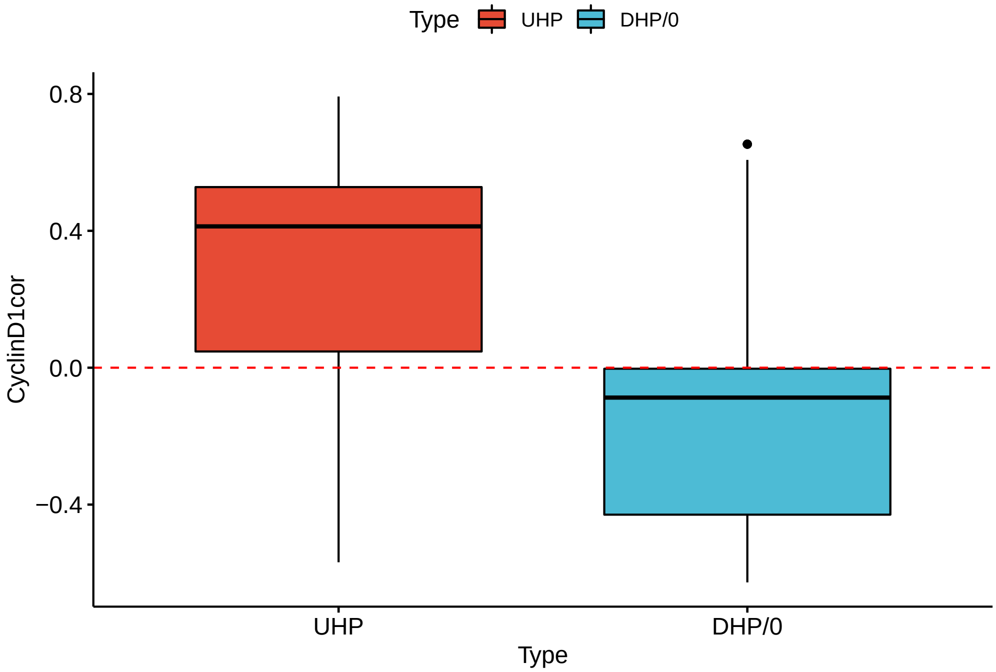

Figure S7: Pearson correlation between PSI of UHD/DHP/Type 0 exons and Cyclin D1 gene expression in the GTEx dataset. Pearson correlation between the expression levels of Cyclin D1 and the PSI of every exon was computed across all GTEx tissues that were examined in this study. Cyclin D1 expression is a proxy for the level of mitosis. As thew figure shows, UHP exons are mostly positively correlated with Cyclin D1 expression, and other exons are mostly negatively correlated.

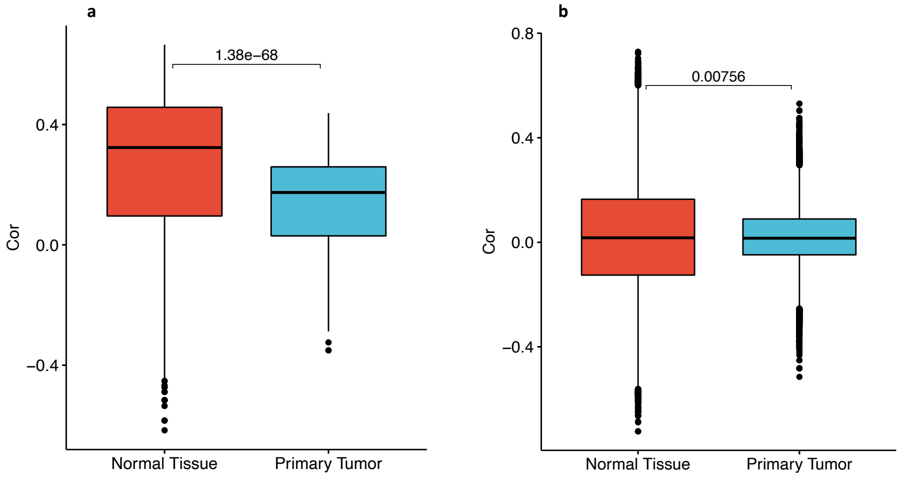

Figure S8: Pearson correlation between PSI of UHD/Type 0 exons and Cyclin D1 gene expression in The Cancer Genome Atlas transcript expression dataset. Pearson correlation between the expression levels of Cyclin D1 and the PSI of every exon was computed in TCGA samples classified as 'Thyroid Carcinoma', separately in the 'Solid Tissue Normal' and 'Primary Tumor' sub-categories, for UHP exons (a) and Type 0 exons (b). UHP exons are mostly positively correlated with Cyclin D1 expression, and Type 0 exons have a median correlation of approximately 0. In tumor the correlation is significantly reduced compared to healthy tissue (Mann-Whitney p-values displayed in the figure), with the gap being significantly larger for UHP exons (for UHP medians of 0.32 and 0.17 for normal and tumor tissues, respectively, and for Type 0 exons medians of 0.017 and 0.016, for normal and tumor tissues, respectively).

Tissue	#Samples
Adrenal Gland	258
Brain - Cortex	255
Breast - Mammary Tissue	459
Heart - Left Ventricle	432
Liver	226
Lung	578
Pancreas	328
Pituitary	283
Spleen	241
Thyroid	653

Table S1: Organs included in the analysis. The column "#Samples" shows the number of samples used from the GTEx RNA-seq resource (37) for the current analysis.

Dataset	Breast tissue	Left Ventricle	Liver
SRA	SRP301453	SRP237337	SRP326468
DHP/DHP	115	128	14
UHP/UHP	34	179	1
DHP/UHP	7	41	0
UHP/DHP	5	53	0
FET	1.15×10^{-22}	1.54×10^{-26}	6.67×10^{-2}

Table S2: UHP/DHP/type 0 exon analysis on three external datasets. We repeated our analysis of UHP/DHP/type 0 exons on three external datasets from NCBI's sequence read archive (SRA) (48). In all three datasets, most of the overlapping exons were type 0 in both the GTEx and the SRA dataset, and most of the other exons were type 0 in one of the datasets (not shown in the table). Rows such as "DHP/UHP" show the counts of exons that were classified as indicated in the SRA/GTEx datasets. FET: Fischer's Exact Text p-value. These results suggest that there is a significant consistency of exon types across different donor cohorts and experimental procedures.

Table S3: Frequency of occurrence of Transcription Factor Binding Sites (TFBS). See main text for definitions of `type 0`, `UHP`, and `DHP`. The Bonferroni corrected threshold of $\alpha = 0.05$ is 9.11×10^{-5}. *) significant at this threshold.

TFBS	type 0	UHP	DHP	0 vs. UHP	0 vs DHP	UHP vs. DHP
ARNT2 (TFFM0853.1)	9.0%	9.7%	10.5%	0.006970	$p < 10^{-6}$ *	0.008200
ARNTL (TFFM0162.1)	1.7%	2.4%	2.4%	$p < 10^{-6}$ *	$p < 10^{-6}$ *	n.s
ASCL1 (TFFM0131.1)	24.6%	21.6%	22.0%	$p < 10^{-6}$ *	$p < 10^{-6}$ *	n.s
ASCL1 (TFFM0131.2)	27.6%	25.3%	25.5%	$p < 10^{-6}$ *	$p < 10^{-6}$ *	n.s
ASCL1 (TFFM0890.1)	28.2%	25.2%	25.6%	$p < 10^{-6}$ *	$p < 10^{-6}$ *	n.s
ASCL2 (TFFM0440.1)	14.7%	12.5%	13.3%	$p < 10^{-6}$ *	$p < 10^{-6}$ *	0.041738
ATF2 (TFFM0653.1)	6.1%	7.4%	7.2%	$p < 10^{-6}$ *	$p < 10^{-6}$ *	n.s
ATF3 (TFFM0003.2)	5.4%	6.6%	6.3%	$p < 10^{-6}$ *	3.90×10^{-5} *	n.s
ATF4 (TFFM0163.2)	2.2%	2.4%	3.1%	n.s	$p < 10^{-6}$ *	7.90×10^{-5} *
BCL6 (TFFM0006.1)	18.0%	16.1%	16.4%	$p < 10^{-6}$ *	$p < 10^{-6}$ *	n.s
BHLHE22 (TFFM0165.1)	26.2%	23.3%	23.2%	$p < 10^{-6}$ *	$p < 10^{-6}$ *	n.s
BHLHE22 (TFFM0794.1)	26.0%	23.1%	23.2%	$p < 10^{-6}$ *	$p < 10^{-6}$ *	n.s
BHLHE22 (TFFM0892.1)	25.6%	22.6%	22.7%	$p < 10^{-6}$ *	$p < 10^{-6}$ *	n.s
CREB1 (TFFM0012.2)	4.4%	5.5%	5.3%	$p < 10^{-6}$ *	$p < 10^{-6}$ *	n.s
CTCFL (TFFM0133.2)	21.5%	23.8%	23.4%	$p < 10^{-6}$ *	$p < 10^{-6}$ *	n.s
ELF3 (TFFM0170.2)	10.3%	8.5%	8.5%	$p < 10^{-6}$ *	$p < 10^{-6}$ *	n.s
ELF4 (TFFM0472.1)	20.6%	18.6%	20.0%	$p < 10^{-6}$ *	n.s	0.001419
ETS2 (TFFM0858.1)	8.5%	6.9%	6.5%	$p < 10^{-6}$ *	$p < 10^{-6}$ *	n.s
ETV4 (TFFM0173.1)	14.2%	12.3%	12.5%	$p < 10^{-6}$ *	$p < 10^{-6}$ *	n.s
ETV5 (TFFM0480.1)	7.8%	6.9%	6.1%	0.000110	$p < 10^{-6}$ *	0.003721
ETV5 (TFFM0785.1)	4.9%	4.2%	3.7%	7.00×10^{-5} *	$p < 10^{-6}$ *	n.s
ETV6 (TFFM0174.1)	20.0%	18.2%	19.4%	$p < 10^{-6}$ *	n.s	0.006593
FOXA2 (TFFM0036.1)	13.8%	12.7%	11.9%	0.000145	$p < 10^{-6}$ *	0.032277
GABPA (TFFM0039.2)	25.7%	26.7%	28.2%	n.s	$p < 10^{-6}$ *	0.002089
GLIS1 (TFFM0492.1)	12.5%	14.9%	14.8%	$p < 10^{-6}$ *	$p < 10^{-6}$ *	n.s
GMEB2 (TFFM0494.1)	6.1%	7.6%	7.7%	$p < 10^{-6}$ *	$p < 10^{-6}$ *	n.s
HOXA13 (TFFM0504.1)	5.0%	4.4%	3.8%	0.001868	$p < 10^{-6}$ *	0.008876
HOXB13 (TFFM0180.1)	3.2%	2.8%	2.3%	n.s	$p < 10^{-6}$ *	0.006023
HOXC9 (TFFM0047.1)	6.6%	5.6%	5.5%	$p < 10^{-6}$ *	$p < 10^{-6}$ *	n.s
IKZF1 (TFFM0509.1)	17.9%	15.2%	16.2%	$p < 10^{-6}$ *	$p < 10^{-6}$ *	0.016385
IRF8 (TFFM0511.1)	14.4%	12.4%	13.3%	$p < 10^{-6}$ *	0.000216	0.019470
KLF9 (TFFM0827.1)	15.7%	17.6%	16.1%	$p < 10^{-6}$ *	n.s	0.000287
MITF (TFFM0141.2)	7.1%	8.1%	8.6%	5.30×10^{-5} *	$p < 10^{-6}$ *	0.044470
MNT (TFFM0191.1)	7.4%	8.6%	8.7%	$p < 10^{-6}$ *	$p < 10^{-6}$ *	n.s
MZF1 (TFFM0531.1)	8.7%	11.2%	10.8%	$p < 10^{-6}$ *	$p < 10^{-6}$ *	n.s
NEUROD1 (TFFM0143.1)	30.6%	28.4%	29.2%	$p < 10^{-6}$ *	n.s	n.s
NFIC (TFFM0072.1)	6.1%	7.7%	7.6%	$p < 10^{-6}$ *	$p < 10^{-6}$ *	n.s
NFIC (TFFM0072.2)	1.6%	1.0%	1.2%	$p < 10^{-6}$ *	n.s	n.s
NFIX (TFFM0761.1)	18.4%	16.1%	17.1%	$p < 10^{-6}$ *	0.000178	0.013094
NFYB (TFFM0075.1)	5.9%	4.9%	5.7%	$p < 10^{-6}$ *	n.s	0.002848
NKX2-5 (TFFM0076.1)	1.2%	1.7%	1.8%	$p < 10^{-6}$ *	$p < 10^{-6}$ *	n.s

Continued on next page

Table S3 – continued from previous page

TFBS	type 0	UHP	DHP	0 vs. UHP	0 vs DHP	UHP vs. DHP
NR2F1 (TFFM0869.1)	7.7%	6.5%	6.4%	$p < 10^{-6}$ *	$p < 10^{-6}$ *	n.s
NR5A1 (TFFM0543.1)	5.1%	4.1%	4.0%	$p < 10^{-6}$ *	$p < 10^{-6}$ *	n.s
NRF1 (TFFM0082.1)	12.2%	14.3%	15.2%	$p < 10^{-6}$ *	$p < 10^{-6}$ *	0.012840
NRF1 (TFFM0732.1)	12.4%	15.0%	15.0%	$p < 10^{-6}$ *	$p < 10^{-6}$ *	n.s
PLAG1 (TFFM0561.1)	15.2%	17.7%	16.9%	$p < 10^{-6}$ *	$p < 10^{-6}$ *	n.s
PRDM14 (TFFM0987.1)	41.5%	44.5%	44.2%	$p < 10^{-6}$ *	$p < 10^{-6}$ *	n.s
RFX3 (TFFM0791.1)	3.8%	5.0%	4.3%	$p < 10^{-6}$ *	n.s	0.000808
SNAI1 (TFFM0877.1)	5.7%	4.1%	4.1%	$p < 10^{-6}$ *	$p < 10^{-6}$ *	n.s
SNAI2 (TFFM0203.1)	13.5%	11.5%	11.4%	$p < 10^{-6}$ *	$p < 10^{-6}$ *	n.s
SOX3 (TFFM0734.1)	8.3%	7.6%	7.1%	0.002438	$p < 10^{-6}$ *	n.s
SP1 (TFFM0097.2)	39.2%	37.9%	36.8%	n.s	$p < 10^{-6}$ *	n.s
SP5 (TFFM0985.1)	4.6%	3.4%	4.1%	$p < 10^{-6}$ *	0.002486	0.004002
SPI1 (TFFM0099.1)	20.0%	18.1%	20.1%	$p < 10^{-6}$ *	n.s	4.70×10^{-5} *
SPIB (TFFM0204.1)	15.0%	13.7%	16.0%	5.60×10^{-5} *	0.001336	$p < 10^{-6}$ *
SPIB (TFFM0204.2)	11.8%	9.8%	10.9%	$p < 10^{-6}$ *	0.002175	0.001131
STAT2 (TFFM0207.1)	5.2%	5.8%	6.2%	0.002084	$p < 10^{-6}$ *	n.s
STAT2 (TFFM0593.1)	8.0%	7.1%	6.8%	0.000223	$p < 10^{-6}$ *	n.s
TCF12 (TFFM0736.1)	4.1%	3.1%	3.3%	$p < 10^{-6}$ *	$p < 10^{-6}$ *	n.s
TCF3 (TFFM0108.1)	25.1%	22.3%	22.3%	$p < 10^{-6}$ *	$p < 10^{-6}$ *	n.s
TCF4 (TFFM0601.1)	16.1%	14.3%	14.1%	$p < 10^{-6}$ *	$p < 10^{-6}$ *	n.s
TEAD2 (TFFM0153.1)	8.9%	10.2%	9.5%	$p < 10^{-6}$ *	0.015357	0.019089
TFAP2A (TFFM0112.1)	13.3%	11.3%	12.9%	$p < 10^{-6}$ *	n.s	8.70×10^{-5} *
TFAP2B (TFFM0114.1)	11.3%	9.5%	11.7%	$p < 10^{-6}$ *	n.s	$p < 10^{-6}$ *
TFE3 (TFFM0798.1)	7.8%	9.0%	8.9%	$p < 10^{-6}$ *	$p < 10^{-6}$ *	n.s
TFEB (TFFM0768.1)	13.5%	15.2%	15.2%	$p < 10^{-6}$ *	$p < 10^{-6}$ *	n.s
THAP11 (TFFM0608.1)	2.3%	2.8%	3.1%	0.000171	$p < 10^{-6}$ *	n.s
VSX2 (TFFM0775.1)	5.5%	4.4%	4.8%	$p < 10^{-6}$ *	0.000384	n.s
YY1 (TFFM0124.1)	11.2%	13.2%	13.3%	$p < 10^{-6}$ *	$p < 10^{-6}$ *	n.s
YY1 (TFFM0714.1)	6.2%	7.6%	7.9%	$p < 10^{-6}$ *	$p < 10^{-6}$ *	n.s
YY2 (TFFM0621.2)	17.5%	19.7%	19.2%	$p < 10^{-6}$ *	$p < 10^{-6}$ *	n.s
ZBTB6 (TFFM0624.1)	32.7%	30.6%	30.2%	$p < 10^{-6}$ *	$p < 10^{-6}$ *	n.s
ZFP42 (TFFM0695.1)	9.3%	12.0%	10.7%	$p < 10^{-6}$ *	$p < 10^{-6}$ *	0.000105
ZIM3 (TFFM0908.1)	3.2%	2.2%	2.2%	$p < 10^{-6}$ *	$p < 10^{-6}$ *	n.s
ZNF135 (TFFM0632.1)	16.9%	15.5%	15.1%	4.50×10^{-5} *	$p < 10^{-6}$ *	n.s
ZNF341 (TFFM0700.1)	14.4%	12.5%	12.5%	$p < 10^{-6}$ *	$p < 10^{-6}$ *	n.s
ZNF417 (TFFM0920.1)	13.7%	15.7%	14.7%	$p < 10^{-6}$ *	0.002549	0.007461
ZNF708 (TFFM0923.1)	5.2%	6.5%	5.9%	$p < 10^{-6}$ *	0.000448	0.013277
ZNF816 (TFFM0914.1)	10.6%	10.2%	9.3%	n.s	$p < 10^{-6}$ *	0.009962
ZNF93 (TFFM0916.1)	19.7%	20.5%	21.6%	n.s	$p < 10^{-6}$ *	0.008743
ATF1 (TFFM0002.1)	13.1%	14.6%	14.2%	$p < 10^{-6}$ *	0.000141	n.s
CTCF (TFFM0014.1)	29.7%	31.6%	30.6%	$p < 10^{-6}$ *	n.s	n.s
E2F1 (TFFM0016.1)	13.7%	14.7%	15.2%	0.000645	$p < 10^{-6}$ *	n.s
ELF1 (TFFM0022.2)	13.5%	12.1%	11.9%	$p < 10^{-6}$ *	$p < 10^{-6}$ *	n.s
ETV4 (TFFM0173.2)	14.7%	13.1%	13.5%	$p < 10^{-6}$ *	0.000197	n.s

Continued on next page

Table S3 – continued from previous page

TFBS	type 0	UHP	DHP	0 vs. UHP	0 vs DHP	UHP vs. DHP
ETV4 (TFFM0784.1)	11.7%	10.5%	10.4%	3.80×10^{-5} *	$p < 10^{-6}$ *	n.s
HSF1 (TFFM0048.1)	4.4%	5.3%	5.0%	$p < 10^{-6}$ *	0.000447	n.s
JUND (TFFM0054.1)	4.5%	5.5%	5.3%	$p < 10^{-6}$ *	1.30×10^{-5} *	n.s
KLF15 (TFFM0515.1)	34.1%	33.8%	32.0%	n.s	$p < 10^{-6}$ *	0.001070
RFX1 (TFFM0733.1)	6.6%	7.8%	7.5%	$p < 10^{-6}$ *	9.80×10^{-5}	n.s
SP4 (TFFM0591.1)	26.5%	25.8%	24.6%	n.s	$p < 10^{-6}$ *	n.s
TFCP2L1 (TFFM0719.1)	17.7%	16.2%	17.4%	$p < 10^{-6}$ *	n.s	0.004856
ZIC3 (TFFM0771.1)	17.7%	19.4%	18.3%	$p < 10^{-6}$ *	n.s	0.008155
ZNF75D (TFFM0647.1)	34.4%	34.2%	32.3%	n.s	$p < 10^{-6}$ *	0.000751
NFYA (TFFM0074.2)	8.4%	8.4%	9.6%	n.s	$p < 10^{-6}$ *	0.000330
PPARG (TFFM0086.1)	8.4%	9.4%	9.6%	3.70×10^{-5} *	$p < 10^{-6}$ *	n.s
SOX9 (TFFM0710.1)	2.8%	2.1%	2.4%	$p < 10^{-6}$ *	n.s	n.s
SPI1 (TFFM0713.1)	14.7%	13.2%	14.2%	$p < 10^{-6}$ *	n.s	0.009807
TBR1 (TFFM0792.1)	1.7%	1.4%	1.2%	n.s	$p < 10^{-6}$ *	n.s
USF2 (TFFM0123.2)	10.8%	12.2%	11.7%	$p < 10^{-6}$ *	0.000614	n.s
ESRRG (TFFM0753.1)	2.3%	1.7%	1.9%	$p < 10^{-6}$ *	n.s	n.s
HOXC9 (TFFM0047.2)	5.8%	5.0%	4.8%	0.000209	$p < 10^{-6}$ *	n.s
NR5A1 (TFFM0871.1)	3.0%	2.3%	2.5%	$p < 10^{-6}$ *	n.s	n.s
FOXH1 (TFFM0037.1)	1.9%	1.4%	1.5%	$p < 10^{-6}$ *	n.s	n.s
TFAP2C (TFFM0211.1)	10.1%	9.7%	8.9%	n.s	$p < 10^{-6}$ *	0.008757
ZNF684 (TFFM0646.1)	1.5%	1.9%	2.0%	n.s	$p < 10^{-6}$ *	n.s
IRF4 (TFFM0182.1)	6.1%	5.2%	5.5%	$p < 10^{-6}$ *	0.000962	n.s
PRDM4 (TFFM0688.1)	7.8%	9.0%	8.6%	$p < 10^{-6}$ *	0.002101	n.s
TCF12 (TFFM0899.1)	11.8%	11.4%	10.5%	n.s	$p < 10^{-6}$ *	0.008528
FLI1 (TFFM0031.1)	31.6%	29.7%	31.9%	$p < 10^{-6}$ *	n.s	5.90×10^{-5} *
NR2C2 (TFFM0079.1)	3.8%	3.4%	3.0%	n.s	$p < 10^{-6}$ *	n.s
BHLHE40 (TFFM0007.1)	13.1%	14.5%	14.1%	$p < 10^{-6}$ *	0.001466	n.s
RFX1 (TFFM0089.1)	5.6%	6.6%	6.0%	1.10×10^{-5} *	n.s	0.028718
TRPS1 (TFFM0980.1)	22.1%	23.7%	23.8%	2.30×10^{-5} *	1.40×10^{-5} *	n.s
CREB1 (TFFM0705.1)	8.0%	9.1%	8.8%	1.50×10^{-5} *	0.000364	n.s
IRF9 (TFFM0757.1)	6.3%	5.4%	5.4%	2.10×10^{-5} *	1.50×10^{-5} *	n.s
MSGN1 (TFFM0862.1)	9.7%	8.6%	9.6%	1.50×10^{-5} *	n.s	0.003044
TFAP2A (TFFM0111.2)	21.0%	19.5%	20.0%	1.50×10^{-5} *	n.s	n.s
ESRRA (TFFM0028.1)	5.7%	6.7%	5.8%	1.60×10^{-5} *	n.s	0.001858
HOXB13 (TFFM0180.2)	2.2%	2.0%	1.7%	n.s	1.70×10^{-5} *	n.s
GATA1 (TFFM0040.1)	3.7%	4.5%	4.2%	1.80×10^{-5} *	n.s	n.s
TCF4 (TFFM0601.2)	23.2%	21.7%	21.6%	7.30×10^{-5} *	1.80×10^{-5} *	n.s
MZF1 (TFFM0531.2)	5.2%	6.1%	5.4%	1.90×10^{-5} *	n.s	0.007048
MAFK (TFFM0058.3)	8.5%	7.5%	7.5%	2.10×10^{-5} *	5.30×10^{-5} *	n.s
THRB (TFFM0885.1)	2.0%	1.9%	1.5%	n.s	2.10×10^{-5} *	n.s
KLF4 (TFFM0056.2)	35.1%	35.3%	33.3%	n.s	2.20×10^{-5} *	0.000394
USF2 (TFFM0123.1)	12.3%	13.5%	12.8%	2.40×10^{-5} *	n.s	0.032162
KLF6 (TFFM0518.1)	19.4%	20.9%	20.5%	2.50×10^{-5} *	0.001505	n.s
ZNF317 (TFFM0639.1)	3.4%	3.8%	4.1%	n.s	2.60×10^{-5} *	n.s

Continued on next page

Table S3 – continued from previous page

TFBS	type 0	UHP	DHP	0 vs. UHP	0 vs DHP	UHP vs. DHP
OVOL2 (TFFM0548.1)	1.4%	1.9%	1.3%	n.s	n.s	2.70×10^{-5} *
RFX1 (TFFM0089.2)	6.5%	7.5%	7.1%	2.70×10^{-5} *	0.008550	n.s
AR (TFFM0001.1)	20.1%	18.7%	19.8%	2.80×10^{-5} *	n.s	0.011240
JUNB (TFFM0829.1)	1.8%	2.3%	2.2%	2.90×10^{-5} *	n.s	n.s
MITF (TFFM0141.1)	10.8%	12.0%	11.3%	3.50×10^{-5} *	n.s	0.043905
TBX20 (TFFM0766.1)	10.0%	10.1%	8.9%	n.s	3.50×10^{-5} *	0.000815
USF1 (TFFM0122.1)	14.1%	15.4%	14.7%	3.50×10^{-5} *	n.s	n.s
NR3C1 (TFFM0080.1)	6.7%	5.8%	5.9%	3.70×10^{-5} *	5.90×10^{-5} *	n.s
HSF2 (TFFM0786.1)	6.9%	7.8%	7.8%	9.60×10^{-5}	3.90×10^{-5} *	n.s
PRDM1 (TFFM0087.2)	4.9%	4.9%	4.2%	n.s	4.10×10^{-5} *	0.002962
HNF1A (TFFM0989.1)	16.8%	17.3%	18.1%	n.s	4.30×10^{-5} *	n.s
OTX2 (TFFM0197.1)	3.2%	2.7%	2.6%	n.s	4.40×10^{-5} *	n.s
ERF (TFFM0476.1)	17.2%	15.9%	16.8%	4.70×10^{-5} *	n.s	0.021609
KLF1 (TFFM0055.1)	19.5%	20.4%	18.6%	n.s	n.s	4.90×10^{-5} *
ZBTB7B (TFFM0770.1)	5.3%	6.1%	5.8%	5.40×10^{-5} *	0.007816	n.s
STAT3 (TFFM0102.1)	10.4%	10.8%	9.3%	n.s	0.000102	6.30×10^{-5} *
KLF4 (TFFM0056.3)	36.7%	38.0%	35.8%	n.s	n.s	6.80×10^{-5} *
IRF2 (TFFM0181.1)	3.6%	3.4%	3.0%	n.s	6.90×10^{-5} *	n.s
MAFG (TFFM0188.2)	5.7%	4.9%	5.0%	7.00×10^{-5} *	0.001299	n.s
NR5A2 (TFFM0731.1)	2.9%	2.4%	2.4%	0.000255	7.90×10^{-5} *	n.s
MAZ (TFFM0524.1)	10.1%	11.2%	10.8%	8.00×10^{-5} *	0.011105	n.s
ESRRB (TFFM0029.1)	3.8%	3.2%	3.3%	8.40×10^{-5} *	0.000604	n.s
MAFF (TFFM0057.2)	4.8%	4.8%	5.6%	n.s	9.10×10^{-5} *	0.000954
ATF7 (TFFM0164.1)	6.1%	6.9%	6.5%	9.80×10^{-5}	n.s	n.s
CREB1 (TFFM0012.1)	16.7%	17.7%	18.0%	0.002328	9.80×10^{-5}	n.s
CEBPG (TFFM0893.1)	7.7%	8.3%	8.7%	0.010044	0.000103	n.s
LHX2 (TFFM0185.2)	3.6%	3.0%	3.3%	0.000104	n.s	n.s
CDX2 (TFFM0008.2)	4.1%	4.8%	4.6%	0.000110	n.s	n.s
ZEB1 (TFFM0127.1)	26.7%	25.2%	25.8%	0.000112	n.s	n.s
TFAP2C (TFFM0793.1)	21.7%	22.8%	23.1%	n.s	0.000114	n.s
SCRT1 (TFFM0580.2)	10.0%	9.1%	9.0%	0.000356	0.000116	n.s
RELB (TFFM0575.1)	4.3%	3.8%	3.7%	0.002573	0.000125	n.s
FOXO3 (TFFM0488.1)	13.3%	12.2%	12.2%	0.000148	0.000258	n.s
ZKSCAN1 (TFFM0630.1)	2.5%	2.9%	3.0%	n.s	0.000150	n.s
SPI1 (TFFM0099.2)	13.9%	12.7%	14.0%	0.000157	n.s	0.001355
KLF12 (TFFM0514.1)	26.2%	26.7%	24.8%	n.s	0.000159	0.000221
HOXD13 (TFFM0808.1)	8.2%	8.9%	7.7%	0.004871	0.015241	0.000164
NEUROD2 (TFFM0990.1)	10.2%	10.3%	11.2%	n.s	0.000170	0.007087
ERG (TFFM0725.1)	21.6%	20.3%	21.7%	0.000175	n.s	0.002476
THAP11 (TFFM0883.1)	13.4%	12.3%	13.1%	0.000185	n.s	0.020255
MYF5 (TFFM0676.1)	17.3%	16.1%	16.4%	0.000191	n.s	n.s
ZSCAN29 (TFFM0648.1)	8.1%	7.3%	7.3%	0.000307	0.000199	n.s
JUN (TFFM0050.1)	10.9%	12.0%	11.4%	0.000214	n.s	n.s
SRF (TFFM0100.1)	4.7%	5.4%	4.5%	0.000215	n.s	0.000642

Continued on next page

Table S3 – continued from previous page

TFBS	type 0	UHP	DHP	0 vs. UHP	0 vs DHP	UHP vs. DHP
EGR1 (TFFM0020.2)	23.0%	24.4%	23.0%	0.000219	n.s	0.002962
GATA4 (TFFM0043.1)	4.7%	5.5%	5.3%	0.000221	0.001686	n.s
PAX5 (TFFM0084.2)	17.6%	18.9%	18.5%	0.000228	0.005266	n.s
ZNF692 (TFFM0984.1)	14.5%	14.2%	13.4%	n.s	0.000228	0.030029
GATA6 (TFFM0137.2)	5.6%	6.4%	6.2%	0.000246	0.003164	n.s
CTCFL (TFFM0133.1)	22.6%	23.9%	23.9%	0.000345	0.000255	n.s
USF2 (TFFM0738.1)	9.6%	10.6%	10.4%	0.000258	0.003336	n.s
ZIC2 (TFFM0628.1)	3.0%	2.4%	2.5%	0.000260	n.s	n.s
NFIC (TFFM0863.1)	8.7%	9.3%	8.1%	0.007537	0.018181	0.000281
NFE2L2 (TFFM0071.1)	6.9%	7.7%	7.6%	0.000285	0.000627	n.s
RUNX3 (TFFM0093.1)	5.5%	5.9%	5.0%	n.s	0.006045	0.000297
SP1 (TFFM0097.1)	23.5%	24.3%	22.5%	n.s	n.s	0.000302
ZBTB7A (TFFM0126.2)	22.1%	21.4%	20.9%	n.s	0.000310	n.s
MAFK (TFFM0058.2)	5.7%	5.1%	6.0%	0.001346	n.s	0.000328
CTCF (TFFM0461.1)	20.5%	21.8%	20.2%	0.000337	n.s	0.000580
BHLHA15 (TFFM0856.1)	7.7%	6.9%	7.1%	0.000339	0.004142	n.s
KLF5 (TFFM0183.1)	26.3%	26.4%	24.9%	n.s	0.000345	0.003945
NFATC1 (TFFM0751.1)	6.8%	7.6%	7.1%	0.000352	n.s	0.036032
NR6A1 (TFFM0544.1)	7.9%	7.0%	7.1%	0.000352	0.000794	n.s
TFAP2C (TFFM0117.1)	13.8%	12.8%	13.8%	0.000355	n.s	0.005043
STAT6 (TFFM0105.1)	9.3%	10.3%	9.9%	0.000395	0.018147	n.s
ZNF257 (TFFM0909.1)	19.8%	21.0%	20.6%	0.000395	n.s	n.s
SOX17 (TFFM0587.1)	16.0%	15.6%	14.9%	n.s	0.000399	n.s
IRF1 (TFFM0708.1)	3.8%	3.5%	3.2%	n.s	0.000404	n.s
MAFB (TFFM0187.1)	4.8%	5.1%	5.5%	n.s	0.000430	n.s
MYOD1 (TFFM0068.2)	14.0%	12.9%	13.4%	0.000431	n.s	n.s
TBX19 (TFFM0597.1)	15.8%	14.7%	14.7%	0.001034	0.000437	n.s
MAFG (TFFM0188.1)	6.0%	5.9%	6.8%	n.s	0.000453	0.001107
MXI1 (TFFM0142.1)	6.6%	7.4%	7.2%	0.000481	0.007622	n.s
NR2C1 (TFFM0542.1)	4.2%	4.3%	3.6%	n.s	0.000486	0.003941
ZNF549 (TFFM0921.1)	20.2%	21.3%	21.4%	0.002466	0.000489	n.s
SPDEF (TFFM0592.1)	8.2%	8.1%	7.4%	n.s	0.000528	0.019320
TCF7 (TFFM0209.2)	6.4%	6.8%	7.1%	n.s	0.000531	n.s
RFX3 (TFFM0577.1)	7.2%	8.0%	7.7%	0.000532	0.010058	n.s
TBP (TFFM0106.1)	5.3%	5.9%	6.0%	0.007525	0.000569	n.s
PTF1A (TFFM0567.1)	17.6%	16.7%	16.5%	n.s	0.000584	n.s
ZNF189 (TFFM0918.1)	19.7%	20.1%	18.6%	n.s	0.000700	0.000628
MAFF (TFFM0057.1)	12.6%	11.6%	12.3%	0.000651	n.s	0.042332
HOXD13 (TFFM0506.1)	8.8%	9.7%	8.9%	0.000717	n.s	0.016009
SCRT1 (TFFM0580.1)	9.9%	9.1%	9.0%	0.001167	0.000721	n.s
SREBF1 (TFFM0797.1)	5.3%	5.1%	5.9%	n.s	0.000730	0.002175
FOXJ2 (TFFM0175.1)	14.1%	13.6%	13.1%	n.s	0.000747	n.s
SNAI2 (TFFM0203.2)	6.0%	5.3%	5.8%	0.000762	n.s	n.s
RBPJ (TFFM0149.1)	6.0%	5.3%	5.7%	0.000772	n.s	n.s

Continued on next page

TFBS	type 0	UHP	DHP	0 vs. UHP	0 vs DHP	UHP vs. DHP
USF2 (TFFM0123.3)	10.3%	11.1%	11.2%	0.002157	0.000773	n.s
ZBTB26 (TFFM0623.1)	18.7%	18.5%	19.8%	n.s	0.000785	0.002750
GATA2 (TFFM0041.2)	3.8%	4.4%	4.4%	0.000835	0.001841	n.s
NFATC2 (TFFM0720.1)	19.0%	19.1%	20.1%	n.s	0.000911	0.017779
PKNOX1 (TFFM0560.2)	4.2%	3.7%	4.5%	n.s	n.s	0.000981
MAF (TFFM0523.1)	24.1%	22.9%	23.8%	0.000984	n.s	n.s
ZNF281 (TFFM0637.1)	18.3%	19.4%	19.3%	0.001003	0.001663	n.s
TBX5 (TFFM0599.1)	4.5%	5.1%	4.6%	0.001016	n.s	0.017567
MEIS1 (TFFM0894.1)	5.1%	4.5%	4.7%	0.001044	n.s	n.s
ZNF652 (TFFM0702.1)	5.3%	5.9%	5.7%	0.001052	n.s	n.s
TFAP2C (TFFM0118.1)	16.4%	17.5%	17.0%	0.001085	n.s	n.s
SOX10 (TFFM0152.1)	13.0%	12.1%	12.1%	0.001363	0.001096	n.s
TP63 (TFFM0120.1)	8.0%	8.8%	8.2%	0.001129	n.s	0.030882
MEF2B (TFFM0189.1)	12.1%	13.0%	12.2%	0.001134	n.s	0.025587
NR2F1 (TFFM0194.1)	3.5%	3.6%	3.0%	n.s	0.001255	0.008668
ELF1 (TFFM0022.1)	18.7%	17.6%	17.8%	0.001298	n.s	n.s
NFYA (TFFM0074.1)	3.2%	2.9%	3.5%	n.s	n.s	0.001303
ZNF449 (TFFM0701.1)	19.2%	19.7%	18.2%	n.s	n.s	0.001344
EBF1 (TFFM0019.2)	4.9%	4.5%	5.2%	n.s	n.s	0.001498
MLX (TFFM0527.1)	4.4%	4.5%	5.0%	n.s	0.001559	n.s
ZBTB18 (TFFM0622.1)	14.2%	13.2%	13.6%	0.001605	n.s	n.s
ISL1 (TFFM0512.1)	13.8%	12.9%	13.8%	0.001675	n.s	0.012928
HNF4G (TFFM0046.1)	4.0%	3.4%	3.8%	0.001676	n.s	n.s
ELK4 (TFFM0024.1)	13.2%	12.9%	14.1%	n.s	0.003207	0.001687
NEUROD2 (TFFM0760.1)	14.7%	13.7%	14.6%	0.001714	n.s	0.028355
GATA1 (TFFM0040.2)	3.8%	4.4%	4.2%	0.001775	n.s	n.s
PRDM1 (TFFM0087.3)	9.6%	10.1%	9.0%	0.028021	0.026310	0.001798
ELF5 (TFFM0718.1)	17.0%	16.0%	16.2%	0.001855	n.s	n.s
SMAD4 (TFFM0151.1)	3.7%	4.0%	4.2%	n.s	0.001878	n.s
SP9 (TFFM0878.1)	3.9%	3.4%	3.8%	0.001881	n.s	n.s
ZNF343 (TFFM0910.1)	12.6%	13.5%	13.3%	0.001959	0.005886	n.s
NEUROD2 (TFFM0533.1)	13.3%	12.4%	13.1%	0.002018	n.s	0.047582
POU2F2 (TFFM0085.1)	12.4%	11.9%	11.6%	n.s	0.002080	n.s
TCF12 (TFFM0107.1)	15.5%	14.6%	14.7%	0.002230	0.006327	n.s
FOXJ3 (TFFM0801.1)	9.9%	9.3%	9.2%	0.011124	0.002298	n.s
EHF (TFFM0471.2)	17.9%	17.6%	18.9%	n.s	0.002397	0.003895
OLIG2 (TFFM0763.1)	6.0%	5.5%	5.4%	0.008476	0.002543	n.s
YY2 (TFFM0621.1)	11.5%	12.3%	11.8%	0.002661	n.s	n.s
RFX5 (TFFM0090.1)	6.2%	6.8%	6.8%	0.002692	0.004805	n.s
CREB5 (TFFM0800.1)	4.3%	4.8%	4.7%	0.002748	n.s	n.s
TFAP2B (TFFM0115.1)	15.4%	15.1%	16.3%	n.s	0.004641	0.002827
MAFG (TFFM0759.1)	10.8%	10.0%	10.5%	0.002829	n.s	n.s
MEIS1 (TFFM0062.1)	10.5%	11.3%	10.9%	0.002831	n.s	n.s
TCF3 (TFFM0737.1)	11.9%	11.4%	11.1%	n.s	0.002893	n.s

Continued on next page

Table S3 – continued from previous page

TFBS	type 0	UHP	DHP	0 vs. UHP	0 vs DHP	UHP vs. DHP
SP2 (TFFM0098.2)	15.6%	14.6%	14.8%	0.003062	0.009185	n.s
ELF5 (TFFM0473.1)	24.0%	23.0%	24.4%	n.s	n.s	0.003274
TBX6 (TFFM0880.1)	12.1%	12.5%	13.0%	n.s	0.003319	n.s
PTF1A (TFFM0888.1)	9.6%	9.0%	8.9%	0.011872	0.003373	n.s
ELF3 (TFFM0170.1)	12.8%	12.0%	12.0%	0.003439	0.004782	n.s
TFCP2 (TFFM0604.1)	8.8%	9.2%	9.6%	n.s	0.003589	n.s
REL (TFFM0715.1)	13.5%	13.8%	14.4%	n.s	0.003612	n.s
WT1 (TFFM0620.1)	4.3%	4.6%	3.9%	n.s	n.s	0.004064
ERG (TFFM0025.1)	24.0%	22.8%	24.2%	n.s	n.s	0.004167
POU2F1 (TFFM0788.1)	13.6%	12.7%	13.2%	0.004268	n.s	n.s
SOX17 (TFFM0711.1)	4.9%	4.7%	5.4%	n.s	n.s	0.004549
FOXA1 (TFFM0035.1)	11.7%	11.2%	10.9%	n.s	0.004596	n.s
DLX5 (TFFM0857.1)	7.2%	7.0%	6.6%	n.s	0.004615	n.s
ETV2 (TFFM0479.1)	25.0%	23.8%	25.2%	n.s	n.s	0.004648
CEBPD (TFFM0011.1)	13.3%	12.9%	12.5%	n.s	0.005008	n.s
POU5F1 (TFFM0148.1)	10.6%	11.3%	11.2%	0.005319	0.012313	n.s
TEAD1 (TFFM0210.1)	6.0%	5.6%	6.3%	n.s	n.s	0.006337
MYCN (TFFM0067.2)	12.8%	13.6%	13.5%	0.006348	0.014029	n.s
ZBTB7A (TFFM0126.1)	5.3%	5.9%	5.7%	0.006676	n.s	n.s
PKNOX1 (TFFM0560.1)	5.3%	4.8%	5.4%	0.006826	n.s	0.008376
OLIG2 (TFFM0991.1)	3.8%	3.6%	4.2%	n.s	n.s	0.006938
BCL6 (TFFM0006.2)	7.0%	7.6%	7.0%	0.007097	n.s	0.029799
GFI1B (TFFM0044.1)	7.9%	7.3%	7.3%	0.008345	0.007182	n.s
MYCN (TFFM0067.1)	8.5%	7.9%	8.0%	0.007806	0.017610	n.s
RFX3 (TFFM0577.2)	8.4%	9.0%	8.4%	0.007861	n.s	0.028152
CRX (TFFM0013.1)	10.8%	11.5%	11.1%	0.008212	n.s	n.s
CRX (TFFM0723.1)	13.6%	14.4%	13.8%	0.008256	n.s	n.s
NFIL3 (TFFM0539.1)	10.3%	11.0%	10.7%	0.008745	n.s	n.s
BCL6B (TFFM0447.1)	11.6%	11.3%	10.9%	n.s	0.009060	n.s
TFAP2B (TFFM0116.1)	14.7%	15.3%	15.5%	n.s	0.009219	n.s
ZNF136 (TFFM0633.1)	5.5%	6.0%	5.6%	0.009226	n.s	n.s
GATA2 (TFFM0041.1)	5.7%	6.3%	6.2%	0.009236	n.s	n.s
ELK1 (TFFM0023.1)	13.6%	14.0%	14.4%	n.s	0.009694	n.s
TCF21 (TFFM0600.1)	15.5%	14.7%	15.6%	0.010412	n.s	0.019447
BHLHE41 (TFFM0450.1)	11.7%	12.4%	12.2%	0.010570	n.s	n.s
MYOD1 (TFFM0068.1)	11.6%	10.9%	11.2%	0.010642	n.s	n.s
ZNF331 (TFFM0919.1)	11.1%	10.5%	11.4%	0.021488	n.s	0.011104
MXI1 (TFFM0142.2)	5.9%	6.4%	6.2%	0.011695	n.s	n.s
ATOH1 (TFFM0854.1)	10.5%	9.9%	9.9%	0.012599	0.012021	n.s
NFYC (TFFM0682.1)	5.1%	4.9%	5.5%	n.s	n.s	0.013208
USF1 (TFFM0122.2)	11.7%	12.4%	12.1%	0.013422	n.s	n.s
PBX2 (TFFM0146.1)	5.7%	5.3%	5.9%	n.s	n.s	0.013657
RFX2 (TFFM0576.1)	7.0%	7.5%	7.5%	0.013825	n.s	n.s
NFKB2 (TFFM0193.1)	9.9%	10.5%	9.7%	0.015750	n.s	0.013931

Continued on next page

Table S3 – continued from previous page

TFBS	type 0	UHP	DHP	0 vs. UHP	0 vs DHP	UHP vs. DHP
BACH2 (TFFM0855.1)	7.4%	7.1%	6.9%	n.s	0.014747	n.s
THRB (TFFM0609.1)	10.4%	9.8%	10.1%	0.015256	n.s	n.s
NFATC1 (TFFM0535.1)	13.7%	14.4%	14.2%	0.016400	n.s	n.s
HOXA13 (TFFM0756.1)	5.9%	6.1%	5.5%	n.s	n.s	0.016432
NFYB (TFFM0075.2)	7.0%	7.1%	7.5%	n.s	0.017033	n.s
HEY2 (TFFM0755.1)	8.9%	9.1%	9.4%	n.s	0.017042	n.s
NR2F6 (TFFM0776.1)	15.8%	16.3%	15.4%	n.s	n.s	0.019455
GRHL2 (TFFM0138.1)	9.1%	9.7%	9.3%	0.020826	n.s	n.s
TP73 (TFFM0121.1)	11.0%	11.2%	10.5%	n.s	n.s	0.030064
SOX2 (TFFM0095.1)	8.8%	8.4%	9.1%	n.s	n.s	0.030714
NFE2L1 (TFFM0192.1)	15.4%	15.2%	16.1%	n.s	n.s	0.032053
ATOH1 (TFFM0004.1)	11.9%	11.6%	12.3%	n.s	n.s	0.032501
NR2F2 (TFFM0144.1)	15.5%	15.6%	14.8%	n.s	n.s	0.035768
EOMES (TFFM0171.1)	12.0%	11.5%	12.2%	n.s	n.s	0.036098
IRF5 (TFFM0852.1)	11.2%	11.0%	11.7%	n.s	n.s	0.039727
TFAP4 (TFFM0212.1)	9.7%	9.4%	10.0%	n.s	n.s	0.049575
TWIST1 (TFFM0155.2)	10.2%	9.7%	10.3%	n.s	n.s	0.08
ARNT (TFFM0161.1)	6.6%	6.7%	6.5%	n.s	n.s	n.s
ATF3 (TFFM0003.1)	11.3%	11.8%	11.6%	n.s	n.s	n.s
ATF4 (TFFM0163.1)	6.0%	6.4%	6.1%	n.s	n.s	n.s
BACH1 (TFFM0654.1)	4.7%	5.0%	4.7%	n.s	n.s	n.s
BACH1 (TFFM0891.1)	3.9%	4.1%	4.0%	n.s	n.s	n.s
BACH2 (TFFM0132.1)	6.8%	6.7%	6.6%	n.s	n.s	n.s
BACH2 (TFFM0132.2)	11.8%	11.4%	11.6%	n.s	n.s	n.s
BATF3 (TFFM0446.1)	0.5%	0.3%	0.5%	n.s	n.s	n.s
BATF3 (TFFM0656.1)	5.9%	5.7%	5.6%	n.s	n.s	n.s
BATF (TFFM0655.1)	6.7%	6.6%	6.8%	n.s	n.s	n.s
BHLHA15 (TFFM0449.1)	13.1%	12.5%	13.0%	n.s	n.s	n.s
BHLHA15 (TFFM0745.1)	6.5%	6.1%	6.2%	n.s	n.s	n.s
CDX2 (TFFM0008.1)	2.6%	2.3%	2.4%	n.s	n.s	n.s
CEBPA (TFFM0009.1)	8.9%	9.0%	8.9%	n.s	n.s	n.s
CEBPA (TFFM0009.2)	9.9%	10.1%	10.4%	n.s	n.s	n.s
CEBPB (TFFM0010.1)	8.8%	9.1%	8.8%	n.s	n.s	n.s
CEBPB (TFFM0722.1)	6.9%	7.0%	6.9%	n.s	n.s	n.s
CEBPD (TFFM0011.2)	10.0%	10.1%	10.3%	n.s	n.s	n.s
CEBPE (TFFM0455.1)	13.1%	12.9%	12.7%	n.s	n.s	n.s
CEBPE (TFFM0799.1)	10.0%	10.2%	9.9%	n.s	n.s	n.s
CEBPG (TFFM0166.1)	7.3%	7.5%	7.2%	n.s	n.s	n.s
CLOCK (TFFM0459.1)	12.5%	12.3%	12.0%	n.s	n.s	n.s
CLOCK (TFFM0795.1)	1.3%	1.2%	1.2%	n.s	n.s	n.s
CREB3L1 (TFFM0167.1)	8.3%	7.9%	8.3%	n.s	n.s	n.s
CREB3L2 (TFFM0746.1)	4.3%	4.4%	4.3%	n.s	n.s	n.s
CREM (TFFM0168.1)	13.7%	14.3%	13.9%	n.s	n.s	n.s
CREM (TFFM0747.1)	7.8%	7.8%	7.8%	n.s	n.s	n.s

Continued on next page

Table S3 – continued from previous page

TFBS	type 0	UHP	DHP	0 vs. UHP	0 vs DHP	UHP vs. DHP
CUX1 (TFFM0169.1)	2.2%	2.0%	2.1%	n.s	n.s	n.s
CUX1 (TFFM0781.1)	8.6%	8.6%	8.5%	n.s	n.s	n.s
CUX2 (TFFM0782.1)	3.3%	3.2%	3.1%	n.s	n.s	n.s
DLX1 (TFFM0804.1)	10.7%	10.2%	10.3%	n.s	n.s	n.s
DLX2 (TFFM0805.1)	7.9%	7.8%	7.6%	n.s	n.s	n.s
DMRT1 (TFFM0464.1)	7.7%	8.0%	7.7%	n.s	n.s	n.s
DUX4 (TFFM0015.1)	2.2%	2.0%	2.1%	n.s	n.s	n.s
E2F4 (TFFM0017.1)	38.2%	40.1%	39.9%	n.s	n.s	n.s
E2F4 (TFFM0017.2)	36.1%	37.4%	36.8%	n.s	n.s	n.s
E2F6 (TFFM0018.1)	10.3%	10.5%	10.6%	n.s	n.s	n.s
E2F6 (TFFM0724.1)	11.0%	11.0%	10.5%	n.s	n.s	n.s
EBF1 (TFFM0019.1)	13.9%	13.5%	13.3%	n.s	n.s	n.s
EBF3 (TFFM0662.1)	24.8%	25.0%	24.7%	n.s	n.s	n.s
EGR1 (TFFM0020.1)	23.4%	24.1%	23.0%	n.s	n.s	n.s
EGR1 (TFFM0020.3)	16.8%	16.7%	16.3%	n.s	n.s	n.s
EGR2 (TFFM0021.1)	15.8%	16.4%	16.3%	n.s	n.s	n.s
EHF (TFFM0471.1)	17.4%	17.4%	17.2%	n.s	n.s	n.s
ELK3 (TFFM0474.1)	13.6%	13.3%	13.4%	n.s	n.s	n.s
ESR1 (TFFM0026.1)	29.9%	30.0%	30.4%	n.s	n.s	n.s
ESR2 (TFFM0027.1)	23.7%	23.5%	23.0%	n.s	n.s	n.s
ESRRA (TFFM0028.2)	1.9%	1.8%	1.6%	n.s	n.s	n.s
ETS1 (TFFM0030.1)	22.2%	21.1%	22.1%	n.s	n.s	n.s
ETV1 (TFFM0172.1)	20.6%	19.9%	20.3%	n.s	n.s	n.s
ETV1 (TFFM0172.2)	35.5%	37.2%	36.0%	n.s	n.s	n.s
ETV5 (TFFM0480.2)	9.5%	10.0%	9.8%	n.s	n.s	n.s
FOS (TFFM0032.1)	8.0%	8.1%	8.1%	n.s	n.s	n.s
FOSL1 (TFFM0033.1)	5.1%	5.4%	5.4%	n.s	n.s	n.s
FOSL1 (TFFM0033.2)	5.3%	5.6%	5.5%	n.s	n.s	n.s
FOSL2 (TFFM0034.1)	6.5%	6.5%	6.3%	n.s	n.s	n.s
FOXA1 (TFFM0035.2)	3.0%	2.8%	2.7%	n.s	n.s	n.s
FOXA2 (TFFM0036.2)	7.5%	7.3%	7.2%	n.s	n.s	n.s
FOXA3 (TFFM0667.1)	10.8%	10.3%	10.6%	n.s	n.s	n.s
FOXF1 (TFFM0486.1)	11.6%	11.8%	12.2%	n.s	n.s	n.s
FOXF2 (TFFM0706.1)	10.8%	11.0%	11.3%	n.s	n.s	n.s
FOXG1 (TFFM0749.1)	12.9%	12.3%	12.7%	n.s	n.s	n.s
FOXK1 (TFFM0134.1)	21.4%	21.4%	20.9%	n.s	n.s	n.s
FOXK2 (TFFM0135.1)	6.4%	6.3%	6.2%	n.s	n.s	n.s
FOXK2 (TFFM0135.2)	5.0%	5.0%	4.8%	n.s	n.s	n.s
FOXO1 (TFFM0038.1)	7.4%	7.6%	7.7%	n.s	n.s	n.s
FOXO3 (TFFM0721.1)	8.7%	9.0%	8.8%	n.s	n.s	n.s
FOXP1 (TFFM0136.1)	3.2%	3.1%	3.0%	n.s	n.s	n.s
FOXP1 (TFFM0726.1)	4.4%	4.9%	4.8%	n.s	n.s	n.s
GABPA (TFFM0039.1)	17.1%	16.7%	17.4%	n.s	n.s	n.s
GATA3 (TFFM0042.1)	1.9%	1.8%	1.8%	n.s	n.s	n.s

Continued on next page

Table S3 – continued from previous page

TFBS	type 0	UHP	DHP	0 vs. UHP	0 vs DHP	UHP vs. DHP
GATA3 (TFFM0042.2)	3.4%	3.4%	3.3%	n.s	n.s	n.s
GATA3 (TFFM0707.1)	3.0%	3.0%	3.0%	n.s	n.s	n.s
GATA4 (TFFM0043.2)	3.0%	3.3%	3.3%	n.s	n.s	n.s
GATA6 (TFFM0137.1)	4.4%	4.7%	4.5%	n.s	n.s	n.s
GFI1 (TFFM0491.1)	3.7%	3.3%	3.3%	n.s	n.s	n.s
GLI2 (TFFM0778.1)	39.1%	39.7%	40.2%	n.s	n.s	n.s
GLI3 (TFFM0859.1)	0.5%	0.5%	0.4%	n.s	n.s	n.s
GLIS2 (TFFM0493.1)	0.3%	0.2%	0.2%	n.s	n.s	n.s
GLIS3 (TFFM0779.1)	2.3%	1.8%	1.9%	n.s	n.s	n.s
GMEB1 (TFFM0750.1)	12.0%	12.0%	11.6%	n.s	n.s	n.s
GRHL1 (TFFM0754.1)	8.1%	8.0%	8.5%	n.s	n.s	n.s
GRHL2 (TFFM0138.2)	9.3%	9.3%	9.3%	n.s	n.s	n.s
HES1 (TFFM0826.1)	0.5%	0.7%	0.8%	n.s	n.s	n.s
HEY1 (TFFM0796.1)	1.7%	1.6%	1.6%	n.s	n.s	n.s
HIF1A (TFFM0139.1)	32.1%	32.6%	32.1%	n.s	n.s	n.s
HLF (TFFM0500.1)	5.6%	5.4%	5.2%	n.s	n.s	n.s
HLF (TFFM0500.2)	5.1%	4.9%	4.9%	n.s	n.s	n.s
HMBOX1 (TFFM0177.1)	0.9%	1.2%	0.9%	n.s	n.s	n.s
HNF1A (TFFM0503.1)	4.7%	4.7%	4.5%	n.s	n.s	n.s
HNF1B (TFFM0178.1)	4.5%	4.5%	4.5%	n.s	n.s	n.s
HNF4A (TFFM0045.1)	3.1%	2.7%	2.7%	n.s	n.s	n.s
HNF4A (TFFM0045.2)	1.7%	1.6%	1.6%	n.s	n.s	n.s
HNF4A (TFFM0860.1)	1.3%	1.3%	1.3%	n.s	n.s	n.s
HNF4G (TFFM0727.1)	2.2%	2.1%	2.1%	n.s	n.s	n.s
HOXA9 (TFFM0179.1)	1.6%	1.6%	1.5%	n.s	n.s	n.s
HOXA9 (TFFM0179.2)	0.5%	0.5%	0.5%	n.s	n.s	n.s
HOXB4 (TFFM0505.1)	8.0%	7.6%	7.9%	n.s	n.s	n.s
HOXB5 (TFFM0806.1)	15.1%	15.7%	15.7%	n.s	n.s	n.s
HOXB8 (TFFM0861.1)	0.4%	0.4%	0.4%	n.s	n.s	n.s
HOXC10 (TFFM0807.1)	8.4%	8.5%	8.1%	n.s	n.s	n.s
IRF1 (TFFM0049.1)	6.0%	6.1%	6.5%	n.s	n.s	n.s
IRF3 (TFFM0851.1)	4.0%	3.8%	3.7%	n.s	n.s	n.s
JUN (TFFM0051.1)	6.2%	6.3%	6.2%	n.s	n.s	n.s
JUN (TFFM0728.1)	3.2%	3.4%	3.2%	n.s	n.s	n.s
JUNB (TFFM0052.1)	4.7%	4.8%	4.7%	n.s	n.s	n.s
JUNB (TFFM0052.2)	4.9%	5.0%	5.0%	n.s	n.s	n.s
JUND (TFFM0053.1)	5.2%	5.3%	5.2%	n.s	n.s	n.s
JUND (TFFM0053.2)	3.3%	3.4%	3.2%	n.s	n.s	n.s
KLF12 (TFFM0780.1)	33.6%	33.1%	32.9%	n.s	n.s	n.s
KLF15 (TFFM0942.1)	2.9%	2.5%	2.5%	n.s	n.s	n.s
KLF16 (TFFM0516.1)	20.9%	21.8%	21.1%	n.s	n.s	n.s
KLF1 (TFFM0729.1)	26.0%	25.8%	25.9%	n.s	n.s	n.s
KLF3 (TFFM0517.1)	39.6%	41.1%	41.0%	n.s	n.s	n.s
KLF4 (TFFM0056.1)	28.4%	28.3%	28.8%	n.s	n.s	n.s

Continued on next page

Table S3 – continued from previous page

TFBS	type 0	UHP	DHP	0 vs. UHP	0 vs DHP	UHP vs. DHP
KLF9 (TFFM0140.1)	21.6%	21.8%	20.8%	n.s	n.s	n.s
LEF1 (TFFM0184.1)	6.1%	5.9%	5.8%	n.s	n.s	n.s
LHX2 (TFFM0185.1)	1.7%	1.4%	1.6%	n.s	n.s	n.s
LHX3 (TFFM0717.1)	2.0%	1.9%	2.1%	n.s	n.s	n.s
LHX6 (TFFM0758.1)	9.2%	9.0%	9.2%	n.s	n.s	n.s
LMX1B (TFFM0772.1)	5.1%	5.3%	5.0%	n.s	n.s	n.s
MAFF (TFFM0730.1)	7.5%	7.5%	7.4%	n.s	n.s	n.s
MAFK (TFFM0058.1)	16.4%	16.1%	16.3%	n.s	n.s	n.s
MAX (TFFM0059.1)	11.1%	11.2%	10.8%	n.s	n.s	n.s
MEF2A (TFFM0060.1)	9.2%	9.3%	9.2%	n.s	n.s	n.s
MEF2A (TFFM0060.2)	9.3%	9.2%	9.5%	n.s	n.s	n.s
MEF2C (TFFM0061.1)	15.0%	15.7%	15.6%	n.s	n.s	n.s
MEF2D (TFFM0525.1)	10.4%	10.5%	10.6%	n.s	n.s	n.s
MEIS2 (TFFM0190.1)	2.5%	3.0%	2.8%	n.s	n.s	n.s
MEIS2 (TFFM0895.1)	6.4%	6.1%	6.1%	n.s	n.s	n.s
MGA (TFFM0526.1)	11.6%	11.6%	11.2%	n.s	n.s	n.s
MYB (TFFM0064.1)	13.2%	13.3%	13.5%	n.s	n.s	n.s
MYB (TFFM0064.2)	8.5%	8.6%	8.3%	n.s	n.s	n.s
MYBL2 (TFFM0065.1)	3.9%	4.0%	4.0%	n.s	n.s	n.s
MYC (TFFM0066.1)	17.4%	17.7%	17.3%	n.s	n.s	n.s
MYC (TFFM0066.2)	6.0%	6.1%	5.6%	n.s	n.s	n.s
MYOG (TFFM0069.1)	8.1%	7.8%	8.0%	n.s	n.s	n.s
MYOG (TFFM0069.2)	14.0%	13.6%	14.1%	n.s	n.s	n.s
NEUROG2 (TFFM0534.1)	14.0%	13.7%	14.2%	n.s	n.s	n.s
NEUROG2 (TFFM0896.1)	14.7%	14.3%	15.0%	n.s	n.s	n.s
NFE2 (TFFM0070.1)	6.0%	5.8%	5.8%	n.s	n.s	n.s
NFE2L1 (TFFM0536.1)	5.8%	5.7%	6.1%	n.s	n.s	n.s
NFIB (TFFM0681.1)	2.8%	2.4%	2.3%	n.s	n.s	n.s
NFIL3 (TFFM0539.2)	10.4%	10.6%	10.5%	n.s	n.s	n.s
NFIX (TFFM0864.1)	2.8%	2.5%	2.5%	n.s	n.s	n.s
NFKB1 (TFFM0073.1)	3.7%	3.8%	3.4%	n.s	n.s	n.s
NKX2-2 (TFFM0683.1)	0.9%	0.7%	0.5%	n.s	n.s	n.s
NKX2-5 (TFFM0076.2)	2.0%	1.9%	1.6%	n.s	n.s	n.s
NKX2-5 (TFFM0077.1)	0.6%	0.4%	0.5%	n.s	n.s	n.s
NKX3-1 (TFFM0078.1)	5.8%	5.7%	5.4%	n.s	n.s	n.s
NKX3-2 (TFFM0716.1)	5.3%	5.8%	5.3%	n.s	n.s	n.s
NKX6-1 (TFFM0762.1)	2.2%	1.9%	1.9%	n.s	n.s	n.s
NR1D1 (TFFM0865.1)	0.4%	0.4%	0.4%	n.s	n.s	n.s
NR1D2 (TFFM0866.1)	0.7%	0.7%	0.6%	n.s	n.s	n.s
NR1H2 (TFFM0993.1)	11.4%	11.7%	11.8%	n.s	n.s	n.s
NR1H4 (TFFM0828.1)	1.6%	1.6%	1.5%	n.s	n.s	n.s
NR2C2 (TFFM0867.1)	2.7%	2.7%	2.2%	n.s	n.s	n.s
NR2F1 (TFFM0868.1)	6.2%	6.7%	6.6%	n.s	n.s	n.s
NR2F6 (TFFM0195.1)	7.3%	7.0%	7.3%	n.s	n.s	n.s

Continued on next page

Table S3 – continued from previous page

TFBS	type 0	UHP	DHP	0 vs. UHP	0 vs DHP	UHP vs. DHP
NR2F6 (TFFM0870.1)	21.3%	21.5%	21.1%	n.s	n.s	n.s
NR4A1 (TFFM0145.1)	2.5%	2.6%	2.4%	n.s	n.s	n.s
NR4A1 (TFFM0145.2)	1.8%	2.1%	1.7%	n.s	n.s	n.s
NR5A2 (TFFM0081.1)	3.2%	3.1%	2.9%	n.s	n.s	n.s
ONECUT1 (TFFM0196.1)	0.7%	0.8%	0.6%	n.s	n.s	n.s
ONECUT1 (TFFM0196.2)	1.1%	1.1%	1.0%	n.s	n.s	n.s
ONECUT2 (TFFM0546.1)	3.9%	3.5%	3.8%	n.s	n.s	n.s
ONECUT2 (TFFM0783.1)	0.6%	0.6%	0.4%	n.s	n.s	n.s
OSR1 (TFFM0872.1)	0.2%	0.2%	0.2%	n.s	n.s	n.s
OSR2 (TFFM0897.1)	25.4%	25.7%	25.2%	n.s	n.s	n.s
OTX2 (TFFM0197.2)	3.9%	4.2%	4.2%	n.s	n.s	n.s
OVOL1 (TFFM0873.1)	1.5%	1.8%	1.8%	n.s	n.s	n.s
PAX3 (TFFM0787.1)	0.3%	0.3%	0.3%	n.s	n.s	n.s
PAX3 (TFFM0874.1)	4.6%	4.8%	5.0%	n.s	n.s	n.s
PAX5 (TFFM0084.1)	5.6%	5.4%	5.5%	n.s	n.s	n.s
PAX7 (TFFM0550.1)	1.3%	1.3%	1.6%	n.s	n.s	n.s
PAX7 (TFFM0764.1)	2.7%	2.7%	3.1%	n.s	n.s	n.s
PBX1 (TFFM0551.1)	5.5%	5.3%	5.4%	n.s	n.s	n.s
PBX2 (TFFM0146.2)	4.9%	5.2%	5.4%	n.s	n.s	n.s
PBX3 (TFFM0147.1)	5.7%	5.6%	5.4%	n.s	n.s	n.s
PDX1 (TFFM0198.2)	3.6%	3.1%	3.3%	n.s	n.s	n.s
PHOX2A (TFFM0773.1)	2.8%	3.1%	3.2%	n.s	n.s	n.s
PHOX2B (TFFM0554.1)	0.7%	0.7%	0.6%	n.s	n.s	n.s
PHOX2B (TFFM0554.2)	0.6%	0.7%	0.6%	n.s	n.s	n.s
PITX1 (TFFM0559.1)	1.2%	1.1%	0.9%	n.s	n.s	n.s
POU2F3 (TFFM0562.1)	6.6%	6.8%	6.6%	n.s	n.s	n.s
POU2F3 (TFFM0562.2)	7.0%	6.9%	6.9%	n.s	n.s	n.s
POU3F1 (TFFM0789.1)	0.8%	0.8%	0.8%	n.s	n.s	n.s
POU3F2 (TFFM0563.1)	3.2%	3.2%	3.3%	n.s	n.s	n.s
PPARD (TFFM0875.1)	3.5%	4.0%	3.6%	n.s	n.s	n.s
PRDM1 (TFFM0087.1)	5.1%	5.3%	5.6%	n.s	n.s	n.s
PRDM4 (TFFM0898.1)	4.1%	4.3%	4.2%	n.s	n.s	n.s
PROP1 (TFFM0774.1)	4.9%	5.1%	5.1%	n.s	n.s	n.s
PROX1 (TFFM0199.1)	0.1%	0.0%	0.1%	n.s	n.s	n.s
PTF1A (TFFM0887.1)	9.5%	9.1%	9.2%	n.s	n.s	n.s
RARA (TFFM0571.1)	2.6%	2.4%	2.6%	n.s	n.s	n.s
RARA (TFFM0777.1)	0.2%	0.1%	0.1%	n.s	n.s	n.s
RARB (TFFM0802.1)	0.4%	0.3%	0.3%	n.s	n.s	n.s
RARB (TFFM0876.1)	0.3%	0.2%	0.2%	n.s	n.s	n.s
RELA (TFFM0200.1)	4.5%	4.1%	4.3%	n.s	n.s	n.s
REST (TFFM0088.1)	10.3%	10.1%	10.1%	n.s	n.s	n.s
RORA (TFFM0709.1)	0.2%	0.1%	0.1%	n.s	n.s	n.s
RORB (TFFM0830.1)	14.1%	14.4%	14.8%	n.s	n.s	n.s
RORC (TFFM0578.1)	9.8%	9.8%	9.7%	n.s	n.s	n.s

Continued on next page

Table S3 – continued from previous page

TFBS	type 0	UHP	DHP	0 vs. UHP	0 vs DHP	UHP vs. DHP
RUNX1 (TFFM0091.1)	8.3%	7.9%	8.3%	n.s	n.s	n.s
RUNX2 (TFFM0092.1)	25.8%	25.7%	26.1%	n.s	n.s	n.s
RUNX3 (TFFM0093.2)	4.4%	4.3%	3.9%	n.s	n.s	n.s
RXRA (TFFM0094.1)	3.1%	2.8%	2.7%	n.s	n.s	n.s
RXRB (TFFM0579.1)	0.7%	0.7%	0.8%	n.s	n.s	n.s
SCRT2 (TFFM0581.1)	7.3%	7.1%	6.9%	n.s	n.s	n.s
SCRT2 (TFFM0581.2)	6.9%	6.9%	6.5%	n.s	n.s	n.s
SIX2 (TFFM0150.1)	5.2%	5.1%	5.5%	n.s	n.s	n.s
SMAD2 (TFFM0201.1)	5.4%	5.8%	5.6%	n.s	n.s	n.s
SMAD3 (TFFM0202.1)	0.2%	0.1%	0.2%	n.s	n.s	n.s
SOX11 (TFFM0803.1)	5.4%	5.5%	5.0%	n.s	n.s	n.s
SOX13 (TFFM0586.1)	5.8%	6.1%	5.9%	n.s	n.s	n.s
SOX2 (TFFM0095.2)	7.6%	7.6%	7.3%	n.s	n.s	n.s
SOX3 (TFFM0096.1)	9.8%	9.6%	9.8%	n.s	n.s	n.s
SOX6 (TFFM0588.1)	3.0%	2.6%	2.6%	n.s	n.s	n.s
SP1 (TFFM0712.1)	30.0%	31.1%	30.5%	n.s	n.s	n.s
SP2 (TFFM0735.1)	27.5%	28.1%	27.3%	n.s	n.s	n.s
SP3 (TFFM0590.1)	18.6%	19.0%	18.5%	n.s	n.s	n.s
SP4 (TFFM0765.1)	26.6%	27.5%	26.7%	n.s	n.s	n.s
SREBF1 (TFFM0205.1)	7.0%	6.8%	6.9%	n.s	n.s	n.s
SREBF1 (TFFM0206.1)	6.5%	6.3%	6.3%	n.s	n.s	n.s
STAT1 (TFFM0101.1)	6.5%	6.6%	6.2%	n.s	n.s	n.s
STAT4 (TFFM0103.1)	13.6%	13.5%	13.4%	n.s	n.s	n.s
STAT5A (TFFM0594.1)	7.7%	7.3%	7.3%	n.s	n.s	n.s
STAT5B (TFFM0595.1)	9.3%	9.2%	9.3%	n.s	n.s	n.s
TBX21 (TFFM0208.1)	3.9%	3.7%	3.5%	n.s	n.s	n.s
TBX21 (TFFM0767.1)	0.3%	0.2%	0.2%	n.s	n.s	n.s
TBX3 (TFFM0598.1)	3.9%	4.1%	3.7%	n.s	n.s	n.s
TBX3 (TFFM0879.1)	0.5%	0.5%	0.5%	n.s	n.s	n.s
TCF21 (TFFM0881.1)	32.4%	31.7%	32.9%	n.s	n.s	n.s
TCF7 (TFFM0209.1)	1.9%	2.0%	1.7%	n.s	n.s	n.s
TCF7L2 (TFFM0109.1)	2.5%	2.4%	2.3%	n.s	n.s	n.s
TEAD1 (TFFM0210.2)	2.8%	2.7%	2.8%	n.s	n.s	n.s
TEAD3 (TFFM0603.1)	1.2%	1.2%	1.3%	n.s	n.s	n.s
TEAD4 (TFFM0110.1)	16.5%	15.9%	16.1%	n.s	n.s	n.s
TEAD4 (TFFM0110.2)	1.8%	1.9%	2.1%	n.s	n.s	n.s
TFAP2A (TFFM0111.1)	18.1%	17.9%	17.5%	n.s	n.s	n.s
TFAP2A (TFFM0113.1)	17.3%	17.4%	17.5%	n.s	n.s	n.s
TFAP4 (TFFM0882.1)	9.3%	8.9%	9.0%	n.s	n.s	n.s
TFCP2 (TFFM0929.1)	3.5%	3.7%	3.7%	n.s	n.s	n.s
TFDP1 (TFFM0154.1)	6.6%	6.6%	6.8%	n.s	n.s	n.s
TFE3 (TFFM0605.1)	7.6%	7.6%	7.9%	n.s	n.s	n.s
TGIF1 (TFFM0606.1)	17.2%	17.8%	17.9%	n.s	n.s	n.s
TGIF2 (TFFM0790.1)	0.5%	0.6%	0.5%	n.s	n.s	n.s

Continued on next page

Table S3 – continued from previous page

TFBS	type 0	UHP	DHP	0 vs. UHP	0 vs DHP	UHP vs. DHP
THAP1 (TFFM0213.1)	2.8%	2.7%	2.8%	n.s	n.s	n.s
THAP1 (TFFM0744.1)	1.8%	2.3%	2.1%	n.s	n.s	n.s
THRB (TFFM0884.1)	6.0%	6.1%	6.2%	n.s	n.s	n.s
TP53 (TFFM0119.1)	2.9%	2.9%	3.0%	n.s	n.s	n.s
TWIST1 (TFFM0155.1)	12.5%	12.0%	12.5%	n.s	n.s	n.s
VDR (TFFM0769.1)	0.2%	0.3%	0.1%	n.s	n.s	n.s
VEZF1 (TFFM0616.1)	19.6%	19.9%	19.4%	n.s	n.s	n.s
XBP1 (TFFM0214.1)	6.0%	5.7%	6.2%	n.s	n.s	n.s
ZBED2 (TFFM0979.1)	4.6%	5.0%	4.8%	n.s	n.s	n.s
ZBTB12 (TFFM0693.1)	3.5%	3.5%	3.7%	n.s	n.s	n.s
ZBTB14 (TFFM0694.1)	0.8%	0.8%	0.7%	n.s	n.s	n.s
ZBTB33 (TFFM0125.1)	3.7%	3.6%	3.7%	n.s	n.s	n.s
ZEB1 (TFFM0127.2)	11.9%	12.1%	12.4%	n.s	n.s	n.s
ZFP57 (TFFM0627.1)	26.8%	28.0%	27.6%	n.s	n.s	n.s
ZFX (TFFM0128.1)	11.2%	11.6%	11.6%	n.s	n.s	n.s
ZIC5 (TFFM0886.1)	23.6%	23.9%	23.2%	n.s	n.s	n.s
ZKSCAN3 (TFFM0981.1)	0.9%	0.9%	0.8%	n.s	n.s	n.s
ZKSCAN5 (TFFM0697.1)	20.9%	20.7%	21.0%	n.s	n.s	n.s
ZNF140 (TFFM0634.1)	1.7%	1.8%	2.0%	n.s	n.s	n.s
ZNF143 (TFFM0129.1)	5.1%	5.6%	5.3%	n.s	n.s	n.s
ZNF148 (TFFM0698.1)	25.1%	25.0%	24.7%	n.s	n.s	n.s
ZNF16 (TFFM0699.1)	0.4%	0.2%	0.3%	n.s	n.s	n.s
ZNF24 (TFFM0156.1)	0.8%	0.7%	0.7%	n.s	n.s	n.s
ZNF263 (TFFM0130.1)	11.3%	11.2%	11.6%	n.s	n.s	n.s
ZNF263 (TFFM0130.2)	7.7%	7.6%	7.5%	n.s	n.s	n.s
ZNF281 (TFFM0889.1)	16.0%	16.1%	16.1%	n.s	n.s	n.s
ZNF282 (TFFM0638.1)	16.3%	16.4%	16.1%	n.s	n.s	n.s
ZNF382 (TFFM0640.1)	1.8%	1.7%	1.7%	n.s	n.s	n.s
ZNF384 (TFFM0157.1)	0.8%	0.6%	0.8%	n.s	n.s	n.s
ZNF416 (TFFM0982.1)	10.7%	10.5%	10.2%	n.s	n.s	n.s
ZNF460 (TFFM0642.1)	3.8%	3.6%	3.5%	n.s	n.s	n.s
ZNF528 (TFFM0643.1)	1.4%	1.5%	1.5%	n.s	n.s	n.s
ZNF582 (TFFM0983.1)	47.4%	48.8%	48.3%	n.s	n.s	n.s
ZNF675 (TFFM0911.1)	3.5%	3.0%	3.2%	n.s	n.s	n.s
ZNF680 (TFFM0922.1)	4.5%	4.7%	4.9%	n.s	n.s	n.s
ZNF682 (TFFM0645.1)	4.6%	4.4%	4.5%	n.s	n.s	n.s
ZNF707 (TFFM0912.1)	10.4%	10.2%	10.4%	n.s	n.s	n.s
ZNF768 (TFFM0924.1)	3.5%	3.6%	3.6%	n.s	n.s	n.s
ZNF85 (TFFM0915.1)	2.4%	2.4%	2.4%	n.s	n.s	n.s
ZNF8 (TFFM0913.1)	4.9%	4.7%	4.6%	n.s	n.s	n.s
ZSCAN4 (TFFM0831.1)	1.0%	0.8%	0.9%	n.s	n.s	n.s

CPE/feature	type 0	UHP	DHP	0 vs. UHP	0 vs DHP	UHP vs. DHP
AnyTags	99.3%	99.9%	99.9%	n.s	n.s	n.s
BREd	16.4%	19.3%	18.1%	0.002699*	0.06	0.18
BREu	15.1%	13.8%	14.6%	0.09	n.s	0.26
Bridge	2.2%	2.3%	2.0%	n.s	n.s	n.s
Broad	90.8%	91.8%	92.1%	n.s	n.s	n.s
CpG	61.3%	65.4%	66.4%	0.000963*	4.20×10^{-5} *	n.s
DCE	2.2%	2.6%	2.4%	n.s	n.s	n.s
DCE3	14.3%	14.3%	13.8%	n.s	n.s	n.s
DPE	12.6%	12.4%	13.0%	n.s	n.s	n.s
Inr	39.7%	40.4%	38.8%	n.s	n.s	n.s
MTE	0.9%	0.8%	0.9%	n.s	n.s	n.s
Sharp	8.6%	8.1%	7.8%	n.s	0.16	n.s
TATA	10.0%	7.4%	6.4%	0.000472*	$p < 10^{-6}$ *	0.15
TCT	12.4%	11.8%	11.3%	n.s	0.10	n.s
XCPE1	3.1%	2.6%	2.4%	n.s	0.08	n.s
XCPE2	2.2%	2.7%	3.0%	n.s	0.036007	n.s

Table S4: Frequency of occurrence of core promoter elements (CPEs) and other promoter-related features defined as in (56). See main text for definitions of `type 0`, `UHP`, and `DHP`. The Bonferroni corrected threshold of α = 0.05 is 3.57×10^{-3}. *) significant at this threshold.

Table S5: Frequency of occurrence of RNA Binding Protein (RBP) Binding Sites. See main text for definitions of `type 0`, `UHP`, and `DHP`. The Bonferroni corrected threshold of α = 0.05 is 7.35×10^{-4}. *) significant at this threshold.

RBP	type 0	UHP	DHP	0 vs. UHP	0 vs DHP	UHP vs. DHP
ELAVL1 (1170_19561594)	48.6%	55.2%	54.4%	$p < 10^{-6}$ *	$p < 10^{-6}$ *	n.s
RBM4 (1172_19561594)	25.0%	20.8%	19.6%	$p < 10^{-6}$ *	$p < 10^{-6}$ *	0.018992
KHDRBS3 (1174_19561594)	30.6%	36.7%	35.5%	$p < 10^{-6}$ *	$p < 10^{-6}$ *	n.s
Vts1 (1176_19561594)	19.7%	17.5%	17.8%	$p < 10^{-6}$ *	$p < 10^{-6}$ *	n.s
YBX1 (1177_19561594)	36.7%	32.8%	32.8%	$p < 10^{-6}$ *	$p < 10^{-6}$ *	n.s
QKI (1215_16041388)	17.9%	20.9%	20.2%	$p < 10^{-6}$ *	$p < 10^{-6}$ *	n.s
ZRANB2 (1285_19304800)	36.3%	40.7%	41.5%	$p < 10^{-6}$ *	$p < 10^{-6}$ *	n.s
HNRNPA1 (23_7510636)	31.0%	33.5%	32.7%	$p < 10^{-6}$ *	9.40×10^{-5} *	n.s
SFRS1 (243_7543047)	4.7%	4.0%	3.5%	9.00×10^{-5} *	$p < 10^{-6}$ *	0.035681
PABPC1 (24_7908267)	20.2%	22.9%	22.7%	$p < 10^{-6}$ *	$p < 10^{-6}$ *	n.s
EIF4B (351_8846295)	29.1%	27.7%	26.4%	0.000237*	$p < 10^{-6}$ *	n.s
a2bp1 (36_12574126)	25.2%	22.9%	23.1%	$p < 10^{-6}$ *	$p < 10^{-6}$ *	n.s
NONO (488_9001221)	41.1%	42.9%	43.6%	n.s	$p < 10^{-6}$ *	n.s
FUS (637_11098054)	28.3%	25.4%	24.7%	$p < 10^{-6}$ *	$p < 10^{-6}$ *	n.s
MBNL1 (669_20071745)	32.0%	29.5%	28.9%	$p < 10^{-6}$ *	$p < 10^{-6}$ *	n.s
ELAVL2 (782_8497264)	5.9%	6.9%	7.2%	1.00×10^{-5} *	$p < 10^{-6}$ *	n.s
ELAVL2 (783_7972035)	10.9%	14.1%	14.2%	$p < 10^{-6}$ *	$p < 10^{-6}$ *	n.s
ELAVL2 (784_7972035)	8.6%	11.2%	10.8%	$p < 10^{-6}$ *	$p < 10^{-6}$ *	n.s
RBMX (922_19282290)	49.6%	45.2%	46.9%	$p < 10^{-6}$ *	$p < 10^{-6}$ *	n.s
PABPC1 (950_7908267)	25.4%	28.2%	28.6%	$p < 10^{-6}$ *	$p < 10^{-6}$ *	n.s
ZFP36 (951_12324455)	7.0%	9.7%	9.3%	$p < 10^{-6}$ *	$p < 10^{-6}$ *	n.s
SFRS1 (242_7543047)	20.7%	20.9%	19.0%	n.s	$p < 10^{-6}$ *	0.000267*
KHSRP (1186_17893325)	9.3%	8.7%	8.1%	0.015073	$p < 10^{-6}$ *	0.06
Pum2 (329_11780640)	8.3%	9.4%	8.9%	$p < 10^{-6}$ *	0.009904	n.s
sap-49 (145_9163526)	18.9%	17.4%	17.9%	1.80×10^{-5} *	0.004345	n.s
Psi (915_11565747)	19.5%	21.0%	20.5%	2.50×10^{-5} *	0.002388	n.s
ybx2-a (114_7499328)	14.2%	15.5%	15.0%	3.50×10^{-5} *	0.008450	n.s
KHDRBS3 (1216_19457263)	3.4%	4.1%	4.0%	0.000121*	0.000766	n.s
ybx2-a (115_7499328)	11.9%	13.0%	12.7%	0.000294*	0.006111	n.s
ZFP36 (221_12324455)	3.3%	3.7%	3.9%	n.s	0.000311*	n.s
SNRPA (662_1717938)	4.1%	4.7%	4.3%	0.000955	n.s	n.s
SFRS1 (952_7543047)	9.7%	9.0%	9.2%	0.002765	n.s	n.s
QKI (149_16041388)	2.3%	2.8%	2.2%	n.s	n.s	0.003777
RBMY1A1 (1052_17318228)	15.3%	14.4%	15.5%	0.005060	n.s	0.009171
SNRPA (946_10094314)	2.9%	3.1%	2.5%	n.s	n.s	0.008204
PTBP1 (1171_19561594)	6.4%	6.9%	6.3%	n.s	n.s	0.039223
NCL (1004_8676391)	0.0%	0.0%	0.0%	n.s	n.s	n.s
NCL (1026_10858445)	5.1%	5.3%	4.9%	n.s	n.s	n.s
RBMY1A1 (1053_17318228)	35.4%	35.1%	36.5%	n.s	n.s	n.s
SFRS13A (1169_19561594)	61.5%	61.1%	61.1%	n.s	n.s	n.s
SFRS1 (1173_19561594)	36.6%	36.1%	34.8%	n.s	n.s	n.s

Continued on next page

Table S5 – continued from previous page

RBP	type 0	UHP	DHP	0 vs. UHP	0 vs DHP	UHP vs. DHP
SNRPA (1175_19561594)	6.6%	6.5%	6.4%	n.s	n.s	n.s
KHSRP (1185_17893325)	1.7%	1.4%	1.6%	n.s	n.s	n.s
ACO1 (1213_8021254)	52.2%	50.9%	50.8%	n.s	n.s	n.s
ybx2-a (130_11376140)	0.3%	0.2%	0.2%	n.s	n.s	n.s
NCL (131_11376140)	1.1%	1.2%	1.2%	n.s	n.s	n.s
KHDRBS3 (147_19457263)	2.9%	3.0%	3.0%	n.s	n.s	n.s
SFRS2 (244_7543047)	11.3%	11.8%	11.4%	n.s	n.s	n.s
sus (254_1714588)	8.9%	9.0%	8.9%	n.s	n.s	n.s
pum (323_16537387)	2.0%	2.4%	2.3%	n.s	n.s	n.s
Pum2 (330_11780640)	1.2%	1.0%	1.2%	n.s	n.s	n.s
EIF4B (350_8846295)	30.4%	30.0%	29.7%	n.s	n.s	n.s
EIF4B (352_8846295)	48.6%	47.9%	47.3%	n.s	n.s	n.s
IGF2BP1 (359_12507992)	0.6%	0.7%	0.6%	n.s	n.s	n.s
Rna15 (376_9199325)	0.3%	0.2%	0.3%	n.s	n.s	n.s
Rna15 (377_9199325)	0.2%	0.1%	0.1%	n.s	n.s	n.s
A2BP1 (37_16537540)	12.4%	12.1%	11.9%	n.s	n.s	n.s
SNRPA (661_1717938)	3.4%	3.8%	3.5%	n.s	n.s	n.s
SNRPA (663_1717938)	2.6%	2.8%	2.7%	n.s	n.s	n.s
NOVA2 (680_9789075)	1.0%	1.1%	0.9%	n.s	n.s	n.s
NOVA2 (682_10811881)	1.4%	1.3%	1.1%	n.s	n.s	n.s
SFRS7 (790_10094314)	2.5%	2.5%	2.6%	n.s	n.s	n.s
SFRS2 (791_10094314)	3.9%	4.3%	4.4%	n.s	n.s	n.s
SFRS9 (797_17548433)	80.1%	80.3%	78.8%	n.s	n.s	n.s
B52 (802_9111335)	0.0%	0.0%	0.0%	n.s	n.s	n.s
SNRPA (947_10094314)	7.1%	6.7%	6.7%	n.s	n.s	n.s
SNRPA (948_10094314)	1.8%	1.8%	1.7%	n.s	n.s	n.s
SNRPA (949_10094314)	32.8%	33.1%	31.6%	n.s	n.s	n.s
SFRS2 (953_7543047)	19.2%	19.6%	18.7%	n.s	n.s	n.s
SFRS7 (954_10094314)	10.2%	10.7%	10.7%	n.s	n.s	n.s
YTHDC1 (969_20167602)	83.0%	81.8%	81.6%	n.s	n.s	n.s

ENCFF012VQA	ENCFF039TJY	ENCFF043FHJ
ENCFF061DKX	ENCFF064ASM	ENCFF065MQK
ENCFF082DKP	ENCFF105LDY	ENCFF107SJD
ENCFF129APS	ENCFF132MQB	ENCFF151LHR
ENCFF159PYD	ENCFF160KVW	ENCFF166EUJ
ENCFF175PDD	ENCFF195PBD	ENCFF211PWC
ENCFF216PEY	ENCFF218GFN	ENCFF226BZE
ENCFF227ELA	ENCFF229KAY	ENCFF235UTX
ENCFF245CAL	ENCFF254MJA	ENCFF258NNN
ENCFF292PBX	ENCFF322DAE	ENCFF341YAZ
ENCFF354VWZ	ENCFF355MNE	ENCFF371CYY
ENCFF376CIT	ENCFF389ZBU	ENCFF394NVG
ENCFF396ZQL	ENCFF410CPY	ENCFF411PMA
ENCFF448ZOJ	ENCFF451XZC	ENCFF456FTV
ENCFF469KDX	ENCFF471ZTS	ENCFF473YGW
ENCFF482BDW	ENCFF493EPF	ENCFF502RVO
ENCFF519DDH	ENCFF535TAL	ENCFF536KQX
ENCFF538YRS	ENCFF540RGI	ENCFF555UBC
ENCFF558UJR	ENCFF567ELA	ENCFF569MJS
ENCFF574UKD	ENCFF607OBG	ENCFF608MSL
ENCFF615EAT	ENCFF616GPO	ENCFF618BDP
ENCFF627GBZ	ENCFF634JRD	ENCFF653SLQ
ENCFF657NRR	ENCFF658XFZ	ENCFF672HZG
ENCFF673HLW	ENCFF673VSN	ENCFF680DFX
ENCFF681FKP	ENCFF681MRC	ENCFF683YQZ
ENCFF698HVQ	ENCFF712JXT	ENCFF730QNU
ENCFF745RUH	ENCFF750UAQ	ENCFF770ENO
ENCFF779EDF	ENCFF834UYS	ENCFF836KEF
ENCFF838LKZ	ENCFF842JME	ENCFF843PJA
ENCFF848IHI	ENCFF869HZM	ENCFF870KJE
ENCFF872EBX	ENCFF880CLF	ENCFF885ZWB
ENCFF886PSD	ENCFF890XQW	ENCFF898ZLY
ENCFF900JDD	ENCFF909CTD	ENCFF918UHB
ENCFF921FKB	ENCFF923UMY	ENCFF946CSJ
ENCFF952WTH	ENCFF967TFR	ENCFF993GPP

Table S6: ChIP-Seq files used in computing the relative binding of RNA Polymerase subunit 2 to exons of types I,II and 0. Each of the entries refers to a BED file downloaded from ENCODE (43).
