# Supplementary material for: Alternative splicing is coupled to gene expression in a subset of variably expressed genes
Source: bioRxiv. 2023 Oct 11:2023.06.13.544742. Originally published 2023 Jun 14. Preprint. [Version 3] doi: 10.1101/2023.06.13.544742 (PMC10312658; doi:10.1101/2023.06.13.544742)
Supplement: Supplement 2 [file media-2.pdf]

term	p.value	Coverage	adj.p	Term Name
GO:0004705	2.04E-54	0.61864407	2.89E-51	JUN kinase activity
GO:0004707	2.22E-54	0.48648649	3.13E-51	MAP kinase activity
GO:0004708	7.68E-50	0.59482759	1.08E-46	MAP kinase kinase activity
GO:0009416	7.90E-44	0.6	1.11E-40	response to light stimulus
GO:0003779	2.78E-35	0.19362187	3.91E-32	actin binding
GO:0038095	2.66E-33	0.50980392	3.74E-30	Fc-epsilon receptor signaling pathway
GO:0042765	6.37E-31	0.65	8.95E-28	GPI-anchor transamidase complex
GO:0007010	7.16E-31	0.31557377	1.01E-27	cytoskeleton organization
GO:0008092	1.30E-30	0.30952381	1.82E-27	cytoskeletal protein binding
GO:0090398	1.02E-29	0.43801653	1.43E-26	cellular senescence
GO:0016255	8.23E-29	0.65454545	1.15E-25	attachment of GPI anchor to protein
GO:1990709	1.10E-28	1	1.54E-25	presynaptic active zone organization
GO:0007254	4.24E-28	0.42857143	5.94E-25	JNK cascade
GO:0010859	1.61E-27	1	2.26E-24	calcium-dependent cysteine-type endopeptidase inhibitor activity
GO:0005739	2.41E-27	0.15780731	3.38E-24	mitochondrion
GO:0051090	6.02E-26	0.48275862	8.41E-23	regulation of DNA-binding transcription factor activity
GO:0006941	1.16E-25	0.79411765	1.62E-22	striated muscle contraction
GO:0006099	7.22E-25	0.44791667	1.01E-21	tricarboxylic acid cycle
GO:0006096	1.51E-24	0.42056075	2.11E-21	glycolytic process
GO:0005200	1.09E-22	0.28703704	1.51E-19	structural constituent of cytoskeleton
GO:0045296	6.22E-22	0.1626575	8.65E-19	cadherin binding
GO:2000675	6.73E-22	0.75	9.36E-19	negative regulation of type B pancreatic cell apoptotic process
GO:0048511	2.01E-21	0.34814815	2.79E-18	rhythmic process
GO:0097340	4.38E-20	0.64102564	6.08E-17	inhibition of cysteine-type endopeptidase activity
GO:0005759	2.67E-19	0.19396552	3.71E-16	mitochondrial matrix
GO:0032559	8.69E-19	0.72413793	1.21E-15	adenyl ribonucleotide binding
GO:0031032	2.55E-18	0.38297872	3.54E-15	actomyosin structure organization
GO:0046514	2.82E-18	0.74074074	3.91E-15	ceramide catabolic process
GO:0009317	3.43E-18	1	4.74E-15	acetyl-CoA carboxylase complex
GO:0019901	1.14E-17	0.15589354	1.58E-14	protein kinase binding
GO:0050810	1.45E-17	0.62857143	2.01E-14	regulation of steroid biosynthetic process
GO:0030913	3.07E-17	0.73076923	4.24E-14	paranodal junction assembly
GO:0005198	4.29E-17	0.21100917	5.93E-14	structural molecule activity
GO:0062098	9.68E-17	0.7037037	1.34E-13	regulation of programmed necrotic cell death
GO:0051287	9.91E-17	0.35714286	1.37E-13	NAD binding
GO:0023051	1.86E-16	0.84210526	2.56E-13	regulation of signaling
GO:0006937	3.26E-16	0.45	4.48E-13	regulation of muscle contraction
GO:0042752	5.79E-16	0.29496403	7.96E-13	regulation of circadian rhythm
GO:0004108	7.04E-16	0.93333333	9.68E-13	citrate (Si)-synthase activity
GO:0004819	3.42E-15	0.76190476	4.69E-12	glutamine-tRNA ligase activity
GO:0007274	6.04E-15	0.57142857	8.28E-12	neuromuscular synaptic transmission
GO:0017064	7.35E-15	0.64285714	1.01E-11	fatty acid amide hydrolase activity
GO:0006425	1.02E-14	0.78947368	1.40E-11	glutaminyl-tRNA aminoacylation
GO:0003723	1.55E-14	0.10666181	2.12E-11	RNA binding
GO:0005743	1.76E-14	0.17118998	2.41E-11	mitochondrial inner membrane
GO:0045261	3.61E-14	0.69565217	4.93E-11	proton-transporting ATP synthase complex, catalytic core F(1)
GO:0051402	6.77E-14	0.30357143	9.25E-11	neuron apoptotic process
GO:0007339	9.80E-14	0.51282051	1.34E-10	binding of sperm to zona pellucida
GO:0030955	1.18E-13	0.77777778	1.61E-10	potassium ion binding
GO:0010807	1.18E-13	0.77777778	1.61E-10	regulation of synaptic vesicle priming
GO:0004743	1.31E-13	0.92307692	1.78E-10	pyruvate kinase activity
GO:0061049	3.19E-13	0.44	4.34E-10	cell growth involved in cardiac muscle cell development
GO:0030182	4.33E-13	0.30188679	5.89E-10	neuron differentiation
GO:0030866	6.24E-13	0.31578947	8.47E-10	cortical actin cytoskeleton organization
GO:0044065	8.57E-13	0.85714286	1.16E-09	regulation of respiratory system process
GO:0070296	8.57E-13	0.85714286	1.16E-09	sarcoplasmic reticulum calcium ion transport
GO:0070972	8.57E-13	0.85714286	1.16E-09	protein localization to endoplasmic reticulum
GO:0004449	9.90E-13	0.65217391	1.34E-09	isocitrate dehydrogenase (NAD+) activity
GO:0005524	1.34E-12	0.10528407	1.81E-09	ATP binding
GO:0046912	1.34E-12	0.76470588	1.82E-09	acyltransferase activity, acyl groups converted into alkyl on transfer
GO:0035516	1.76E-12	0.91666667	2.38E-09	oxidative DNA demethylase activity
GO:0071344	1.76E-12	0.91666667	2.38E-09	diphosphate metabolic process
GO:0086066	1.76E-12	0.91666667	2.38E-09	atrial cardiac muscle cell to AV node cell communication
GO:0030315	1.79E-12	0.45454545	2.42E-09	T-tubule
GO:0106310	2.53E-12	0.16444444	3.40E-09	protein serine kinase activity
GO:0032781	4.67E-12	0.37704918	6.29E-09	positive regulation of ATP-dependent activity
GO:0000226	8.32E-12	0.20425532	1.12E-08	microtubule cytoskeleton organization
GO:0099525	1.07E-11	0.84615385	1.45E-08	presynaptic dense core vesicle exocytosis
GO:0044331	1.20E-11	0.5	1.62E-08	cell-cell adhesion mediated by cadherin
GO:1904813	1.27E-11	0.22404372	1.70E-08	ficolin-1-rich granule lumen
GO:0043202	1.49E-11	0.2556391	1.99E-08	lysosomal lumen
GO:0043531	1.49E-11	0.359375	2.00E-08	ADP binding
GO:0004672	1.60E-11	0.12465116	2.14E-08	protein kinase activity
GO:0016082	1.68E-11	0.46153846	2.25E-08	synaptic vesicle priming
GO:0033539	1.68E-11	0.46153846	2.25E-08	fatty acid beta-oxidation using acyl-CoA dehydrogenase
GO:0015629	1.85E-11	0.16839378	2.47E-08	actin cytoskeleton
GO:0071205	2.27E-11	0.60869565	3.03E-08	protein localization to juxtaparanode region of axon
GO:0002175	2.36E-11	0.90909091	3.16E-08	protein localization to paranode region of axon
GO:0014902	2.60E-11	0.51612903	3.47E-08	myotube differentiation
GO:0099536	2.60E-11	0.51612903	3.47E-08	synaptic signaling
GO:0030018	2.74E-11	0.24	3.65E-08	Z disc
GO:0031013	3.35E-11	1	4.46E-08	troponin I binding
GO:0005915	4.87E-11	0.5	6.48E-08	zonula adherens
GO:0006754	5.09E-11	0.58333333	6.77E-08	ATP biosynthetic process
GO:0046933	5.43E-11	0.53571429	7.22E-08	proton-transporting ATP synthase activity, rotational mechanism
GO:0007043	6.24E-11	0.33823529	8.28E-08	cell-cell junction assembly
GO:0006101	8.84E-11	0.61904762	1.17E-07	citrate metabolic process
GO:0046513	1.40E-10	0.3559322	1.86E-07	ceramide biosynthetic process
GO:0060048	1.40E-10	0.3559322	1.86E-07	cardiac muscle contraction
GO:0005856	1.75E-10	0.14054927	2.32E-07	cytoskeleton
GO:0006102	2.02E-10	0.59090909	2.68E-07	isocitrate metabolic process
GO:0005912	2.72E-10	0.19815668	3.59E-07	adherens junction
GO:0106022	3.14E-10	0.9	4.15E-07	positive regulation of vesicle docking
GO:0071900	3.43E-10	0.63157895	4.52E-07	regulation of protein serine/threonine kinase activity
GO:0098696	3.43E-10	0.63157895	4.52E-07	regulation of neurotransmitter receptor localization to postsynaptic specialization membrane
GO:2001259	3.43E-10	0.63157895	4.52E-07	positive regulation of cation channel activity
GO:0007015	3.49E-10	0.18215613	4.60E-07	actin filament organization
GO:0030507	4.08E-10	0.3	5.37E-07	spectrin binding
GO:0008307	4.12E-10	0.25	5.42E-07	structural constituent of muscle
GO:0002639	4.28E-10	0.51851852	5.62E-07	positive regulation of immunoglobulin production
GO:0051015	4.39E-10	0.14677104	5.75E-07	actin filament binding
GO:0004347	4.89E-10	1	6.41E-07	glucose-6-phosphate isomerase activity
GO:0032972	4.89E-10	1	6.41E-07	regulation of muscle filament sliding speed
GO:0090646	4.89E-10	1	6.41E-07	mitochondrial tRNA processing
GO:0032229	5.41E-10	0.76923077	7.07E-07	negative regulation of synaptic transmission, GABAergic
GO:0106006	8.91E-10	0.54166667	1.16E-06	cytoskeletal protein-membrane anchor activity
GO:0030049	8.91E-10	0.54166667	1.16E-06	muscle filament sliding
GO:0042645	1.04E-09	0.33898305	1.36E-06	mitochondrial nucleoid
GO:0006468	1.04E-09	0.11606392	1.36E-06	protein phosphorylation
GO:0070061	1.08E-09	0.45454545	1.41E-06	fructose binding
GO:0006446	1.50E-09	0.26530612	1.95E-06	regulation of translational initiation
GO:0006120	1.51E-09	0.31818182	1.96E-06	mitochondrial electron transport, NADH to ubiquinone
GO:0016747	1.60E-09	0.36734694	2.08E-06	acyltransferase activity, transferring groups other than amino-acyl groups
GO:0043217	1.62E-09	0.81818182	2.11E-06	myelin maintenance
GO:0031430	1.74E-09	0.52	2.26E-06	M band
GO:0042776	1.74E-09	0.52	2.26E-06	proton motive force-driven mitochondrial ATP synthesis
GO:0030176	2.50E-09	0.1959799	3.24E-06	integral component of endoplasmic reticulum membrane
GO:0016342	2.55E-09	0.46666667	3.30E-06	catenin complex
GO:0001725	2.67E-09	0.22222222	3.46E-06	stress fiber
GO:0008093	2.78E-09	0.30882353	3.60E-06	cytoskeletal anchor activity
GO:0061157	3.00E-09	0.4	3.88E-06	mRNA destabilization
GO:0034604	3.17E-09	0.61111111	4.10E-06	pyruvate dehydrogenase (NAD+) activity
GO:0016651	3.26E-09	0.5	4.20E-06	oxidoreductase activity, acting on NAD(P)H
GO:0004332	3.62E-09	0.54545455	4.66E-06	fructose-bisphosphate aldolase activity
GO:0005764	3.68E-09	0.15789474	4.74E-06	lysosome
GO:0008360	3.73E-09	0.19047619	4.79E-06	regulation of cell shape
GO:0045104	3.85E-09	0.31746032	4.96E-06	intermediate filament cytoskeleton organization
GO:0045259	4.13E-09	0.88888889	5.31E-06	proton-transporting ATP synthase complex
GO:0036371	4.13E-09	0.88888889	5.31E-06	protein localization to T-tubule
GO:0090258	4.13E-09	0.88888889	5.31E-06	negative regulation of mitochondrial fission
GO:0090335	4.13E-09	0.88888889	5.31E-06	regulation of brown fat cell differentiation
GO:0010881	4.77E-09	0.41666667	6.11E-06	regulation of cardiac muscle contraction by regulation of the release of sequestered calcium ion
GO:0048312	4.99E-09	0.66666667	6.39E-06	intracellular distribution of mitochondria
GO:0015631	5.15E-09	0.22142857	6.58E-06	tubulin binding
GO:0003988	5.89E-09	0.48148148	7.52E-06	acetyl-CoA C-acyltransferase activity
GO:1904753	5.89E-09	0.48148148	7.52E-06	negative regulation of vascular associated smooth muscle cell migration
GO:0005515	6.64E-09	0.09007982	8.47E-06	protein binding
GO:0006086	7.07E-09	0.57894737	9.01E-06	acetyl-CoA biosynthetic process from pyruvate
GO:0003989	7.09E-09	0.52173913	9.03E-06	acetyl-CoA carboxylase activity
GO:0012501	7.09E-09	0.52173913	9.03E-06	programmed cell death
GO:0030172	7.14E-09	1	9.08E-06	troponin C binding
GO:0031071	7.14E-09	1	9.08E-06	cysteine desulfurase activity
GO:0047860	7.14E-09	1	9.08E-06	diiodophenylpyruvate reductase activity
GO:0042245	7.14E-09	1	9.08E-06	RNA repair
GO:0098907	7.14E-09	1	9.08E-06	regulation of SA node cell action potential
GO:1990938	7.14E-09	1	9.08E-06	peptidyl-aspartic acid autophosphorylation
GO:0015031	9.30E-09	0.14255765	1.18E-05	protein transport
GO:0055114	9.78E-09	0.13355593	1.24E-05	oxidation-reduction process
GO:0030388	1.16E-08	0.39473684	1.47E-05	fructose 1,6-bisphosphate metabolic process
GO:0003680	1.18E-08	0.42424242	1.49E-05	minor groove of adenine-thymine-rich DNA binding
GO:0035552	1.25E-08	0.625	1.58E-05	oxidative single-stranded DNA demethylation
GO:0008137	1.34E-08	0.32727273	1.69E-05	NADH dehydrogenase (ubiquinone) activity
GO:0098685	1.44E-08	0.25531915	1.81E-05	Schaffer collateral - CA1 synapse
GO:0003211	1.86E-08	0.69230769	2.34E-05	cardiac ventricle formation
GO:0010882	1.86E-08	0.69230769	2.34E-05	regulation of cardiac muscle contraction by calcium ion signaling
GO:0086070	1.94E-08	0.8	2.44E-05	SA node cell to atrial cardiac muscle cell communication

GO:0050750	2.52E-08	0.31578947	3.17E-05	low-density lipoprotein particle receptor binding
GO:0043039	2.65E-08	0.375	3.32E-05	tRNA aminoacylation
GO:0061732	2.84E-08	0.58823529	3.57E-05	mitochondrial acetyl-CoA biosynthetic process from pyruvate
GO:0007268	2.88E-08	0.20666667	3.61E-05	chemical synaptic transmission
GO:1900015	2.90E-08	0.52380952	3.63E-05	regulation of cytokine production involved in inflammatory response
GO:0005829	4.69E-08	0.09137056	5.87E-05	cytosol
GO:0046034	4.73E-08	0.32075472	5.91E-05	ATP metabolic process
GO:0004658	4.88E-08	0.64285714	6.09E-05	propionyl-CoA carboxylase activity
GO:0008308	4.88E-08	0.64285714	6.09E-05	voltage-gated anion channel activity
GO:0003006	4.88E-08	0.64285714	6.09E-05	developmental process involved in reproduction
GO:0019626	4.88E-08	0.64285714	6.09E-05	short-chain fatty acid catabolic process
GO:0005134	5.37E-08	0.875	6.68E-05	interleukin-2 receptor binding
GO:0016615	5.37E-08	0.875	6.68E-05	malate dehydrogenase activity
GO:0045145	5.37E-08	0.875	6.68E-05	single-stranded DNA 5'-3' exodeoxyribonuclease activity
GO:1901019	5.37E-08	0.875	6.68E-05	regulation of calcium ion transmembrane transporter activity
GO:1904428	5.37E-08	0.875	6.68E-05	negative regulation of tubulin deacetylation
GO:1904692	5.37E-08	0.875	6.68E-05	positive regulation of type B pancreatic cell proliferation
GO:0000159	6.31E-08	0.2739726	7.81E-05	protein phosphatase type 2A complex
GO:0003065	6.68E-08	0.72727273	8.27E-05	positive regulation of heart rate by epinephrine
GO:0016078	6.68E-08	0.72727273	8.27E-05	tRNA catabolic process
GO:1902115	6.68E-08	0.72727273	8.27E-05	regulation of organelle assembly
GO:0006936	7.22E-08	0.19512195	8.92E-05	muscle contraction
GO:0045773	8.09E-08	0.27027027	9.98E-05	positive regulation of axon extension
GO:0006635	8.81E-08	0.22727273	0.000108675	fatty acid beta-oxidation
GO:0034551	9.77E-08	0.47826087	0.000120328	mitochondrial respiratory chain complex III assembly
GO:1902807	9.77E-08	0.47826087	0.000120328	negative regulation of cell cycle G1/S phase transition
GO:2001135	9.77E-08	0.47826087	0.000120328	regulation of endocytic recycling
GO:0005865	1.04E-07	1	0.000128027	striated muscle thin filament
GO:1990584	1.04E-07	1	0.000128027	cardiac Troponin complex
GO:0005460	1.04E-07	1	0.000128027	UDP-glucose transmembrane transporter activity
GO:0032549	1.04E-07	1	0.000128027	ribonucleoside binding
GO:0018283	1.04E-07	1	0.000128027	iron incorporation into metallo-sulfur cluster
GO:0032272	1.04E-07	1	0.000128027	negative regulation of protein polymerization
GO:0034111	1.04E-07	1	0.000128027	negative regulation of homotypic cell-cell adhesion
GO:0072684	1.04E-07	1	0.000128027	mitochondrial tRNA 3'-trailer cleavage, endonucleolytic
GO:0050633	1.14E-07	0.6	0.000139781	acetyl-CoA C-myristoyltransferase activity
GO:0070476	1.14E-07	0.6	0.000139781	rRNA (guanine-N7)-methylation
GO:2000553	1.14E-07	0.6	0.000139781	positive regulation of T-helper 2 cell cytokine production
GO:0086005	1.15E-07	0.42857143	0.000140475	ventricular cardiac muscle cell action potential
GO:0031333	1.18E-07	0.30357143	0.000143246	negative regulation of protein-containing complex assembly
GO:0043560	1.19E-07	0.52631579	0.000144592	insulin receptor substrate binding
GO:0060292	1.19E-07	0.52631579	0.000144592	long-term synaptic depression
GO:0086004	1.19E-07	0.52631579	0.000144592	regulation of cardiac muscle cell contraction
GO:0030426	1.36E-07	0.19375	0.000164386	growth cone
GO:0005861	1.47E-07	0.35897436	0.000177562	troponin complex
GO:0072659	1.49E-07	0.1745283	0.000180284	protein localization to plasma membrane
GO:0030674	1.49E-07	0.18934911	0.000180649	protein-macromolecule adaptor activity
GO:0004176	1.69E-07	0.45833333	0.000204405	ATP-dependent peptidase activity
GO:0045214	1.71E-07	0.22641509	0.000206385	sarcomere organization
GO:0097512	1.88E-07	0.66666667	0.000227287	cardiac myofibril
GO:0009134	1.88E-07	0.66666667	0.000227287	nucleoside diphosphate catabolic process
GO:0055012	1.88E-07	0.66666667	0.000227287	ventricular cardiac muscle cell differentiation
GO:0031902	2.09E-07	0.19018405	0.000252038	late endosome membrane
GO:0055010	2.11E-07	0.35	0.000253983	ventricular cardiac muscle tissue morphogenesis
GO:0045176	2.23E-07	0.5	0.000268161	apical protein localization
GO:0060973	2.23E-07	0.5	0.000268161	cell migration involved in heart development
GO:0030060	2.27E-07	0.77777778	0.000272509	L-malate dehydrogenase activity
GO:0032034	2.27E-07	0.77777778	0.000272509	myosin II head/neck binding
GO:0007412	2.27E-07	0.77777778	0.000272509	axon target recognition
GO:0005967	2.46E-07	0.5625	0.000293999	mitochondrial pyruvate dehydrogenase complex
GO:1990829	2.46E-07	0.5625	0.000293999	C-rich single-stranded DNA binding
GO:0032873	2.46E-07	0.5625	0.000293999	negative regulation of stress-activated MAPK cascade
GO:0044805	2.46E-07	0.5625	0.000293999	late nucleophagy
GO:0045761	2.46E-07	0.5625	0.000293999	regulation of adenylate cyclase activity
GO:0051170	2.46E-07	0.5625	0.000293999	import into nucleus
GO:0004674	2.51E-07	0.13319672	0.000299456	protein serine/threonine kinase activity
GO:0008206	2.83E-07	0.44	0.000336881	bile acid metabolic process
GO:0005761	3.01E-07	0.30188679	0.000357559	mitochondrial ribosome
GO:0030216	3.77E-07	0.36111111	0.000448206	keratinocyte differentiation
GO:0016874	4.00E-07	0.2962963	0.00047495	ligase activity
GO:0015986	4.22E-07	0.33333333	0.000500469	proton motive force-driven ATP synthesis
GO:0043274	4.26E-07	0.3125	0.000504582	phospholipase binding
GO:0044224	4.41E-07	0.38709677	0.000521628	juxtaparanode region of axon
GO:0033270	4.60E-07	0.61538462	0.000544055	paranode region of axon
GO:0051560	4.60E-07	0.42307692	0.000544055	mitochondrial calcium ion homeostasis
GO:1902966	4.60E-07	0.61538462	0.000544055	positive regulation of protein localization to early endosome
GO:2000463	4.60E-07	0.42307692	0.000544055	positive regulation of excitatory postsynaptic potential
GO:1900063	4.90E-07	0.52941176	0.000577619	regulation of peroxisome organization
GO:1990504	6.86E-07	0.85714286	0.000808551	dense core granule exocytosis
GO:0035196	6.87E-07	0.45454545	0.000808551	miRNA processing
GO:0042698	6.86E-07	0.85714286	0.000808551	ovulation cycle
GO:0070350	6.86E-07	0.85714286	0.000808551	regulation of white fat cell proliferation
GO:0070407	6.86E-07	0.85714286	0.000808551	oxidation-dependent protein catabolic process
GO:1901021	6.86E-07	0.85714286	0.000808551	positive regulation of calcium ion transmembrane transporter activity
GO:1903296	6.86E-07	0.85714286	0.000808551	positive regulation of glutamate secretion, neurotransmission
GO:0005962	7.12E-07	0.7	0.000833391	mitochondrial isocitrate dehydrogenase complex (NAD+)
GO:0016715	7.12E-07	0.7	0.000833391	oxidoreductase activity, acting on paired donors, with incorporation or reduction of molecular oxygen, reduced ascorbate as one donor, and incorporation of one atom of oxygen
GO:0055069	7.12E-07	0.7	0.000833391	zinc ion homeostasis
GO:0060327	7.12E-07	0.7	0.000833391	cytoplasmic actin-based contraction involved in cell motility
GO:0034452	7.28E-07	0.40740741	0.000849723	dynactin binding
GO:0060307	7.28E-07	0.40740741	0.000849723	regulation of ventricular cardiac muscle cell membrane repolarization
GO:0016020	8.55E-07	0.09134615	0.000995948	membrane
GO:0051893	9.77E-07	0.265625	0.001137385	regulation of focal adhesion assembly
GO:0008802	1.01E-06	0.57142857	0.001172347	betaine-aldehyde dehydrogenase activity
GO:0032754	1.01E-06	0.57142857	0.001172347	positive regulation of interleukin-5 production
GO:0035542	1.01E-06	0.57142857	0.001172347	regulation of SNARE complex assembly
GO:0080111	1.01E-06	0.57142857	0.001172347	DNA demethylation
GO:0086014	1.01E-06	0.57142857	0.001172347	atrial cardiac muscle cell action potential
GO:0006744	1.09E-06	0.33333333	0.001263595	ubiquinone biosynthetic process
GO:0072542	1.12E-06	0.39285714	0.001301208	protein phosphatase activator activity
GO:0046512	1.12E-06	0.39285714	0.001301208	sphingosine biosynthetic process
GO:0060038	1.12E-06	0.39285714	0.001301208	cardiac muscle cell proliferation
GO:0030017	1.47E-06	0.30434783	0.001702042	sarcomere
GO:0005459	1.52E-06	1	0.001752411	UDP-galactose transmembrane transporter activity
GO:0035605	1.52E-06	1	0.001752411	peptidyl-cysteine S-nitrosylase activity
GO:0097109	1.52E-06	1	0.001752411	neuroligin family protein binding
GO:0031536	1.52E-06	1	0.001752411	positive regulation of exit from mitosis
GO:0072386	1.52E-06	1	0.001752411	plus-end-directed organelle transport along microtubule
GO:1900148	1.52E-06	1	0.001752411	negative regulation of Schwann cell migration
GO:1902047	1.52E-06	1	0.001752411	polyamine transmembrane transport
GO:1903772	1.52E-06	1	0.001752411	regulation of viral budding via host ESCRT complex
GO:1904719	1.52E-06	1	0.001752411	positive regulation of AMPA glutamate receptor clustering
GO:0034614	1.55E-06	0.24657534	0.001773413	cellular response to reactive oxygen species
GO:0016616	1.60E-06	0.20168067	0.001831646	oxidoreductase activity, acting on the CH-OH group of donors, NAD or NADP as acceptor
GO:0052548	1.64E-06	0.47368421	0.001873573	regulation of endopeptidase activity
GO:2000727	1.64E-06	0.47368421	0.001873573	positive regulation of cardiac muscle cell differentiation
GO:0005744	1.70E-06	0.37931034	0.00193712	TIM23 mitochondrial import inner membrane translocase complex
GO:0099072	1.70E-06	0.37931034	0.00193712	regulation of postsynaptic membrane neurotransmitter receptor levels
GO:0005791	1.75E-06	0.28301887	0.001996506	rough endoplasmic reticulum
GO:0032456	1.81E-06	0.2345679	0.002059421	endocytic recycling
GO:0009083	1.83E-06	0.41666667	0.002084352	branched-chain amino acid catabolic process
GO:0010940	1.83E-06	0.41666667	0.002084352	positive regulation of necrotic cell death
GO:0003185	1.84E-06	0.63636364	0.002086794	sinoatrial valve morphogenesis
GO:0071374	1.84E-06	0.63636364	0.002086794	cellular response to parathyroid hormone stimulus
GO:0034599	1.89E-06	0.20535714	0.002140414	cellular response to oxidative stress
GO:0098609	1.99E-06	0.14569536	0.002245502	cell-cell adhesion
GO:0031672	2.03E-06	0.53333333	0.002293045	A band
GO:0016435	2.03E-06	0.53333333	0.002293045	rRNA (guanine) methyltransferase activity
GO:0030518	2.03E-06	0.53333333	0.002293045	intracellular steroid hormone receptor signaling pathway
GO:0070989	2.03E-06	0.53333333	0.002293045	oxidative demethylation
GO:0008016	2.43E-06	0.26229508	0.002734143	regulation of heart contraction
GO:0004598	2.58E-06	0.75	0.002907526	peptidylamidoglycolate lyase activity
GO:0005483	2.58E-06	0.75	0.002907526	soluble NSF attachment protein activity
GO:0008592	2.58E-06	0.75	0.002907526	regulation of Toll signaling pathway
GO:0044571	2.58E-06	0.75	0.002907526	[2Fe-2S] cluster assembly
GO:0009098	2.58E-06	0.75	0.002907526	leucine biosynthetic process
GO:0061762	2.58E-06	0.75	0.002907526	CAMKK-AMPK signaling cascade
GO:1902988	2.58E-06	0.75	0.002907526	neurofibrillary tangle assembly
GO:0016491	2.78E-06	0.13013699	0.003103414	oxidoreductase activity
GO:0051258	2.80E-06	0.45	0.003128035	protein polymerization
GO:0047496	2.82E-06	0.30952381	0.003148881	vesicle transport along microtubule
GO:0035050	2.83E-06	0.33333333	0.003154052	embryonic heart tube development
GO:0045202	2.99E-06	0.14634146	0.003329435	synapse
GO:0007165	3.13E-06	0.10049192	0.003488311	signal transduction
GO:0017101	3.66E-06	0.35483871	0.004067494	aminoacyl-tRNA synthetase multienzyme complex
GO:0007269	3.66E-06	0.35483871	0.004067494	neurotransmitter secretion
GO:0001836	3.75E-06	0.26785714	0.004161455	release of cytochrome c from mitochondria
GO:2000643	3.81E-06	0.5	0.004228476	positive regulation of early endosome to late endosome transport
GO:0000814	4.15E-06	0.58333333	0.004601374	ESCRT II complex
GO:2000234	4.15E-06	0.58333333	0.004601374	positive regulation of rRNA processing
GO:0098978	4.27E-06	0.1443299	0.004718612	glutamatergic synapse
GO:0008179	4.38E-06	0.38461538	0.004834408	adenylate cyclase binding
GO:0055117	5.23E-06	0.34375	0.005771391	regulation of cardiac muscle contraction
GO:0048854	5.74E-06	0.2745098	0.006329322	brain morphogenesis
GO:0050714	5.97E-06	0.24615385	0.006576854	positive regulation of protein secretion
GO:0006939	6.65E-06	0.28888889	0.007316597	smooth muscle contraction
GO:0099092	6.77E-06	0.47058824	0.007443267	postsynaptic density, intracellular component

GO:0031630	6.77E-06	0.47058824	0.007443267	regulation of synaptic vesicle fusion to presynaptic active zone membrane
GO:0051983	6.77E-06	0.47058824	0.007443267	regulation of chromosome segregation
GO:0071625	6.77E-06	0.47058824	0.007443267	vocalization behavior
GO:0030141	6.92E-06	0.23287671	0.00758786	secretory granule
GO:0004739	7.30E-06	0.66666667	0.007992648	pyruvate dehydrogenase (acetyl-transferring) activity
GO:0035515	7.30E-06	0.66666667	0.007992648	oxidative RNA demethylase activity
GO:0043532	7.30E-06	0.66666667	0.007992648	angiostatin binding
GO:0002576	7.30E-06	0.66666667	0.007992648	platelet degranulation
GO:0006333	7.30E-06	0.66666667	0.007992648	chromatin assembly or disassembly
GO:0032231	7.30E-06	0.66666667	0.007992648	regulation of actin filament bundle assembly
GO:1903748	7.30E-06	0.66666667	0.007992648	negative regulation of establishment of protein localization to mitochondrion
GO:1904714	7.31E-06	0.40909091	0.007992648	regulation of chaperone-mediated autophagy
GO:0075522	7.35E-06	0.33333333	0.007994779	IRES-dependent viral translational initiation
GO:0006633	7.59E-06	0.22222222	0.008246428	fatty acid biosynthetic process
GO:0007219	7.98E-06	0.21348315	0.008661203	Notch signaling pathway
GO:0016072	8.46E-06	0.53846154	0.00917208	rRNA metabolic process
GO:0035984	8.46E-06	0.53846154	0.00917208	cellular response to trichostatin A
GO:0036309	8.46E-06	0.53846154	0.00917208	protein localization to M-band
GO:0042426	8.46E-06	0.53846154	0.00917208	choline catabolic process
GO:0060081	8.46E-06	0.53846154	0.00917208	membrane hyperpolarization
GO:0086015	8.46E-06	0.53846154	0.00917208	SA node cell action potential
GO:0043564	8.60E-06	0.83333333	0.00926904	Ku70:Ku80 complex
GO:0004748	8.60E-06	0.83333333	0.00926904	ribonucleoside-diphosphate reductase activity, thioredoxin disulfide as acceptor
GO:0031812	8.60E-06	0.83333333	0.00926904	P2Y1 nucleotide receptor binding
GO:0046625	8.60E-06	0.83333333	0.00926904	sphingolipid binding
GO:0004852	8.60E-06	0.83333333	0.00926904	uroporphyrinogen-III synthase activity
GO:0006780	8.60E-06	0.83333333	0.00926904	uroporphyrinogen III biosynthetic process
GO:0009386	8.60E-06	0.83333333	0.00926904	translational attenuation
GO:0010124	8.60E-06	0.83333333	0.00926904	phenylacetate catabolic process
GO:0033108	8.60E-06	0.83333333	0.00926904	mitochondrial respiratory chain complex assembly
GO:0060762	8.60E-06	0.83333333	0.00926904	regulation of branching involved in mammary gland duct morphogenesis
GO:0062112	8.60E-06	0.83333333	0.00926904	fatty acid primary amide biosynthetic process
GO:1904292	8.60E-06	0.83333333	0.00926904	regulation of ERAD pathway
GO:1905166	8.60E-06	0.83333333	0.00926904	negative regulation of lysosomal protein catabolic process
GO:0030673	9.52E-06	0.35714286	0.010136498	axolemma
GO:0016079	9.52E-06	0.35714286	0.010136498	synaptic vesicle exocytosis
GO:0016208	1.12E-05	0.27659574	0.011958212	AMP binding
GO:0070531	1.13E-05	0.39130435	0.011975999	BRCA1-A complex
GO:0016624	1.13E-05	0.39130435	0.011975999	oxidoreductase activity, acting on the aldehyde or oxo group of donors, disulfide as acceptor
GO:0032026	1.13E-05	0.39130435	0.011975999	response to magnesium ion
GO:0005758	1.30E-05	0.21428571	0.013740893	mitochondrial intermembrane space
GO:1902494	1.36E-05	0.34482759	0.014423605	catalytic complex
GO:0019887	1.36E-05	0.34482759	0.014423605	protein kinase regulator activity
GO:0098686	1.40E-05	0.31428571	0.014731418	hippocampal mossy fiber to CA3 synapse
GO:0001947	1.45E-05	0.24193548	0.01530219	heart looping
GO:0042060	1.56E-05	0.17777778	0.016423605	wound healing
GO:0050821	1.57E-05	0.14767932	0.016513938	protein stabilization
GO:0005251	1.59E-05	0.5	0.016742181	delayed rectifier potassium channel activity
GO:0030054	1.62E-05	0.15577889	0.016979538	cell junction
GO:0070062	1.65E-05	0.09348739	0.017306362	extracellular exosome
GO:0031091	1.69E-05	0.375	0.017772207	platelet alpha granule
GO:0001659	1.69E-05	0.375	0.017772207	temperature homeostasis
GO:0032059	1.72E-05	0.6	0.017988002	bleb
GO:0044304	1.72E-05	0.6	0.017988002	main axon
GO:1903135	1.72E-05	0.6	0.017988002	cupric ion binding
GO:0042780	1.72E-05	0.6	0.017988002	tRNA 3'-end processing
GO:0004860	1.78E-05	0.23809524	0.018601713	protein kinase inhibitor activity
GO:0097001	1.86E-05	0.42105263	0.019353926	ceramide binding
GO:0005747	1.99E-05	0.22535211	0.020718534	mitochondrial respiratory chain complex I
GO:0031146	2.18E-05	0.234375	0.022696804	SCF-dependent proteasomal ubiquitin-dependent protein catabolic process
GO:0003824	2.20E-05	0.11952191	0.022826206	catalytic activity
GO:0005886	2.21E-05	0.09145775	0.022945226	plasma membrane
GO:0021987	2.49E-05	0.19791667	0.025846612	cerebral cortex development
GO:0006000	2.52E-05	0.2972973	0.026061008	fructose metabolic process
GO:0048787	2.81E-05	0.46666667	0.029046578	presynaptic active zone membrane
GO:0051222	2.81E-05	0.46666667	0.029046578	positive regulation of protein transport
GO:0014704	2.85E-05	0.27272727	0.029314831	intercalated disc
GO:0004565	2.84E-05	0.71428571	0.029314831	beta-galactosidase activity
GO:0010181	2.85E-05	0.27272727	0.029314831	FMN binding
GO:0022904	2.85E-05	0.27272727	0.029314831	respiratory electron transport chain
GO:0000038	2.85E-05	0.27272727	0.029314831	very long-chain fatty acid metabolic process
GO:0001519	2.84E-05	0.71428571	0.029314831	peptide amidation
GO:0015786	2.84E-05	0.71428571	0.029314831	UDP-glucose transmembrane transport
GO:0040040	2.84E-05	0.71428571	0.029314831	thermosensory behavior
GO:0060770	2.84E-05	0.71428571	0.029314831	negative regulation of epithelial cell proliferation involved in prostate gland development
GO:0098910	2.84E-05	0.71428571	0.029314831	regulation of atrial cardiac muscle cell action potential
GO:1903829	2.84E-05	0.71428571	0.029314831	positive regulation of protein localization
GO:0004866	2.94E-05	0.25490196	0.030050775	endopeptidase inhibitor activity
GO:0046726	3.56E-05	0.54545455	0.036336257	positive regulation by virus of viral protein levels in host cell
GO:0005523	3.63E-05	0.3125	0.037023327	tropomyosin binding
GO:0006515	3.63E-05	0.3125	0.037023327	protein quality control for misfolded or incompletely synthesized proteins
GO:2001016	3.63E-05	0.3125	0.037023327	positive regulation of skeletal muscle cell differentiation
GO:0019905	3.68E-05	0.18691589	0.037436086	syntaxin binding
GO:0043005	4.14E-05	0.13953488	0.042014302	neuron projection
GO:0005975	4.14E-05	0.14112903	0.042014302	carbohydrate metabolic process
GO:0007006	4.42E-05	0.38095238	0.044777489	mitochondrial membrane organization
GO:0048143	4.42E-05	0.38095238	0.044777489	astrocyte activation
GO:0071549	4.57E-05	0.24528302	0.046200166	cellular response to dexamethasone stimulus
GO:0006094	4.62E-05	0.26086957	0.04670055	gluconeogenesis
GO:0086091	4.62E-05	0.26086957	0.04670055	regulation of heart rate by cardiac conduction
GO:0001401	4.69E-05	0.4375	0.047348907	SAM complex
GO:0031674	4.69E-05	0.4375	0.047348907	I band
GO:0045792	4.69E-05	0.4375	0.047348907	negative regulation of cell size
GO:0086046	4.69E-05	0.4375	0.047348907	membrane depolarization during SA node cell action potential
GO:2000987	4.69E-05	0.4375	0.047348907	positive regulation of behavioral fear response
GO:0006891	4.86E-05	0.21052632	0.048746278	intra-Golgi vesicle-mediated transport
GO:1903672	4.89E-05	0.3030303	0.049027708	positive regulation of sprouting angiogenesis
